# Extension of Compositional Space to the Ternary in Alloy Chiral Nanoparticles through Galvanic Replacement Reactions

**DOI:** 10.1002/advs.202001321

**Published:** 2020-10-27

**Authors:** Ziyue Ni, Yuanmin Zhu, Junjun Liu, Lin Yang, Peng Sun, Meng Gu, Zhifeng Huang

**Affiliations:** ^1^ Department of Physics Hong Kong Baptist University (HKBU) Kowloon Tong Kowloon Hong Kong SAR China; ^2^ Department of Materials Science and Engineering Southern University of Science and Technology (SUSTech) Shenzhen 518055 China; ^3^ SUSTech Academy for Advanced Interdisciplinary studies Southern University of Science and Technology (SUSTech) Shenzhen 518055 China; ^4^ HKBU Institute of Research and Continuing Education Shenzhen Guangdong 518057 China; ^5^ Institute of Advanced Materials State Key Laboratory of Environmental and Biological Analysis Golden Meditech Centre for NeuroRegeneration Sciences HKBU Kowloon Tong Kowloon Hong Kong SAR China

**Keywords:** chiral nanoparticles, galvanic replacement reactions, glancing angle deposition, optical activity, ternary alloys

## Abstract

Metal chiral nanoparticles (CNPs), composed of atomically chiral lattices, are an emerging chiral nanomaterial showing unique asymmetric properties. Chirality transmission from the host CNPs mediated with galvanic replacement reactions (GRRs) has been carried out to extend their compositional space from the unary to binary. Further compositional extension to, e.g., the ternary is of fundamental interest and in practical demand. Here, layer‐by‐layer glancing angle deposition is used to dope galvanically “inert” dopant Au in the host Cu CNPs to generate binary Cu:Au CNPs. The “inert” dopants serve as structural scaffold to assist the chirality transmission from the host to the third metals (M: Pt and Ag) cathodically precipitating in the CNPs, enabling the formation of polycrystalline ternary Cu:Au:M CNPs whose compositions are tailored with engineering the GRR duration. More scaffold Au atoms are favored for the faster chirality transfer, and the Au‐assisted chirality transfer follows the first‐order kinetics with the reaction rate coefficient of ≈0.3 h^−1^ at room temperature. This work provides further understanding of the GRR‐mediated chirality transfer and paves the way toward enhancing the application functions in enantiodifferentiation, enantioseperation, asymmetric catalysis, bioimaging, and biodetection.

## Graphical abstract

Galvanic replacement of binary Cu:Au chiral nanoparticles (CNPs), where galvanically “inert” Au serves as structural scaffolds, results in chirality transfer from the binary CNPs to generate ternary Cu:Au:Pt and Cu:Au:Ag CNPs that inherently have optical activity. The Au‐assisted chirality transfer follows first‐order kinetics with a reaction rate coefficient of ≈0.3 h^−1^ at room temperature.

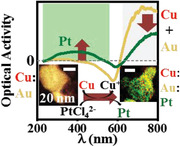

## 1 Introduction

Galvanic replacement reactions (GRRs), denoting the dissolution of metals (serving as sacrificial template) through galvanic oxidation (i.e., anode with low electrode potential) and the concurrent precipitation of second metals onto the sacrificial template through galvanic reduction of precursor metallic cations (i.e., cathode with high electrode potential),^[^ ^1^ ^]^ have enabled controllable engineering of nanoparticles with respect to constituent elements, composition, morphology, surface area, and size.^[^ ^2^ ^]^ Such flexible nanoengineering has been applied to catalysis,^[^ ^3^ ^]^ photoacoustic imaging,^[^ ^4^ ^]^ and disease therapy.^[^ ^5^ ^]^

On the other hand, Chirality, an asymmetric property causing an object to be nonsuperimposable on its mirror image, is a natural phenomenon especially in biological systems that are almost exclusively homochiral, meaning that biomolecules naturally occur in only one of two stereoisomeric configurations.^[^ ^6^ ^]^ It has been of fundamental interest to apply diverse chiral forces to impose structural chirality onto inorganic nanomaterials,^[^ ^7^ ^]^ resulting in novel asymmetric properties that have enabled the fast development of important applications in the fields of light polarization,^[^ ^8^, ^9^ ^]^ chiral sensing,^[^ ^10^ ^]^ refractive index sensing,^[^ ^11^, ^12^ ^]^ nanorheological measurement,^[^ ^13^ ^]^ drug delivery,^[^ ^14^ ^]^ and bioapplications.^[^^]^ Among the diverse approaches developed to generate chiral nanostructures, glancing angle deposition (GLAD) shows the superior features in one‐step generation, production scalability, and device fabrication compatibility.^[^ ^16^ ^]^ GLAD is physical vapor deposition operated at a glancing deposition angle *α* > 80° with respect to the normal direction of a substrate,^[^ ^17^ ^]^ and the consequent self‐shadowing effect leads to the formation of a close‐packed array of tilted nanopillars.^[^ ^18^ ^]^ During GLAD, substrate movement is usually applied to sculpture the shape of the deposited nanopillars,^[^ ^19^ ^]^ and rotating a substrate (at a rate *R*_r_ < 1 degree per second, or ° s^−1^) in clockwise and counterclockwise results in the production of right‐handed (RH) and left‐handed (LH) nanohelices having a characteristic helical pitch (*P*) >50 nm, respectively.^[^ ^20^ ^]^ The helicity at the nanoscale causes inorganic nanostructures to have optical (or chiroptical) activity,^[^ ^9^, ^21^ ^]^ denoting the differential interaction of an object with LH and RH circularly polarized light.^[^ ^22^ ^]^

Furthermore, the health‐associated bioapplications substantially derive from the enantioselective (or enantiospecific) interaction of chiral metamaterials and biomolecules, i.e., a given chiral biomolecule has a differential interaction with LH and RH nanomaterials. However, biomolecules typically possess the sub‐*P* size so that the enantiospecific interaction is usually weak. Recently, we devised GLAD with fast substrate rotation (GLAD‐FSR, at a *R*_r_ of 1–10 ° s^−1^) to reduce *P* to as small as 2 nm, resulting in the formation of metal chiral nanoparticles (CNPs) with the chirality at the atomic scale rather than at the micro/nanoscale.^[^ ^23^, ^24^ ^]^ In the GLAD‐FSR, macroscopic shear forces are applied by substrate rotation along with the translation of incident atoms,^[^ ^25^ ^]^ to produce the CNPs inherently consisting of atomically chiral lattices, including an excess of the twinning lattices, chiral defects in grain boundaries, and the wavelike chiral lattices.^[^ ^26^ ^]^ The chiral lattices make the CNPs chiroptically active and cause the enantiospecific adsorption of chiral molecules on the CNPs,^[^ ^27^ ^]^ leading to the remarked amplification of molecular optical activity^[^ ^28^, ^29^ ^]^ and the enantioselective photoinduced cyclodimerization of 2‐anthracenecarboxylic acid.^[^ ^30^ ^]^

Currently, the CNPs mainly consist of monoelemental (or unary) metals. Multielemental (more than three elements) achiral nanoparticles have been fabricated^[^ ^31^ ^]^ to show remarkedly superior features in catalytic activity, selectivity, and stability compared to the unary counterparts,^[^ ^32^ ^]^ due to the multielement‐induced synergistic effect.^[^ ^33^ ^]^ It intuitively inspires us to explore the extension of compositional space for producing polyelemental CNPs, paving the way toward the functionality enhancement in the chirality‐related applications of enantiodifferentiation (differentiation of a chiral molecule (or enantiomer) from its mirror image), enantioseparation (separation of an enantiomer from its mirror image), bioimaging, biodetection, drug delivery, and single‐enantiomer pharmaceutical production.^[^ ^26^ ^]^ Diverse methods, having been developed to fabricate polyelemental achiral nanoparticles, usually involve high‐temperature or high‐energy alloying,^[^ ^34^ ^]^ which will inevitably lead to the degradation of the chiral lattices that are thermodynamically metastable to achiral lattices.^[^ ^26^ ^]^ Therefore, it is very challenging but of fundamental significance to produce polyelemental CNPs.

In this context, currently we fabricated binary CNPs through the GRR to successfully mediate the chirality transmission (or transfer) from an unary CNPs functioning as chiral sacrificial template (CST) to the binary nanoproducts,^[^ ^35^ ^]^ that is, the galvanically reduced metal atoms precipitate on the CST and replace the CST atoms in a chiral arrangement resembling that of the CST (i.e., the duplication of the CST's atomic chirality). Due to relatively low reduction potential and well‐established nanoparticle synthesis, Ag^[^ ^36^ ^]^ and Cu^[^ ^37^ ^]^ with well‐defined size and shape have been widely used to serve as sacrificial templates in the GRR (Table S1, Supporting Information). We found that Ag CNPs could generally function as the CST for chirality transmission to produce binary CNPs composed of Ag:Au, Ag:Pt, and Ag:Pd.^[^ ^35^ ^]^ By contrast, Cu CNPs could only mediate the chirality transfer to Ag,^[^ ^35^ ^]^ but not to Pt (Figure S1, Supporting Information) or Au (Figure S2, Supporting Information) with unknown reason. It will unavoidably limit the compositional diversity that has a determinant effect on the functionality in the above‐mentioned applications closely associated with molecular chirality.

In this work, a thin layer of metals with high electrode potential (e.g., Au) was doped into the unary Cu CNPs through layer‐by‐layer GLAD (or LbL‐GLAD) to form the binary Cu:Au CNPs.^[^ ^38^ ^]^ The dopant Au, which is resistant to the galvanic oxidation/dissolution in a given electrolyte, serves as structural scaffold to assist the chirality transmission from the CST Cu CNPs mediated with the GRR, leading to the formation of ternary CNPs (such as Cu:Au:Pt, and Cu:Au:Ag). The composition of the ternary CNPs was facilely tuned as a function of GRR duration (*t*). The GRR evolution and ambient aging of the ternary CNPs were studied, and the kinetics of the GRR‐mediated chirality transfer was quantitatively evaluated. Doping the unary CNPs with “inert” metals is a facile approach to extend the compositional space from the binary to ternary in alloy CNPs through the mediation of GRRs, which is in fundamental demand to study the composition‐determined properties and chirality‐associated applications of alloy CNPs.

## 2 Results and Discussion

### 2.1 Binary Cu:Au CNPs (Serving as the CSTs)

**Figure 1:**
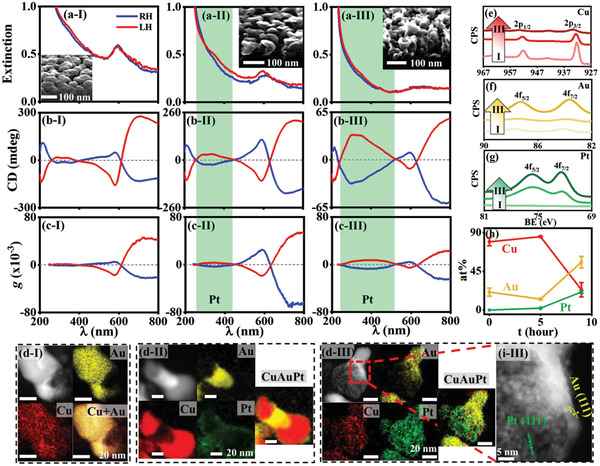
Formation of ternary Cu:Au:Pt CNPs, through the GRR of binary Cu:Au CNPs in an aqueous electrolyte containing 10 µmol L^−1^ K_2_PtCl_4_, as a function of reaction duration (*t*): (I) 0, (II) 5, and (III) 9 h. In the CST binary CNPs, the nominal pitch (*P*) of the host Cu is ≈10 nm and the nominal thickness of Au (*T*_Au_) is 20 nm. The GRR was performed at room temperature through stirring the electrolyte at a rate of 380 rpm, to generate ternary Cu:Au:Pt CNPs. UV/visible spectra: a) extinction, b) CD, c) anisotropic *g*‐factor. a–c) Blue lines: the GRR of right‐handed (RH) binary CNPs; red lines: the GRR of left‐handed (LH) binary CNPs. Green backgrounds are used to mark the LSPR and CD (or chiroplasmonic) signals associated with the precipitating Pt. Insets: (a‐I, a‐II, a‐III) SEM oblique images of the samples. d) HAADF‐STEM images and the corresponding EDS atomic percentage (or at%) mapping of Cu (in red), Au (in yellow), Pt (in green), and their mixtures, as a function of *t* (scale bar: 20 nm). XPS spectra of the binary CNPs treated by the GRR as a function of *t*: e) Cu 2p, f) Au 4f, g) Pt 4f. (e–g) Gradually darkening colors represent an elongation of the GRR from (I) 0 h to (III) 9 h. h) Plot of the mean at% of Cu, Au, and Pt in the CNPs versus *t*, evaluated from the EDS at% mapping of more than five individual CNPs. Error bars represent the standard deviation (SD). (i‐III) HRSTEM image of the portion of the ternary CNP highlighted by red dash square in (d‐III). Insets in (a‐I, II, III) and (d–i): the GRR of the RH‐binary CNPs.

To assist the chirality transmission from the host Cu CNPs mediated with the GRR, Au was doped in the host to generate binary Cu:Au CNPs that will serve as the CSTs to generate ternary CNPs through the GRR. The three‐step LbL‐GLAD was carried out to sandwich an achiral Au layer with a nominal thickness *T*_Au_ of 20 nm between the two host Cu CNPs, each of which had a nominal *P* ≈ 10 nm and *H* ≈ 50 nm. It was formed the binary Cu:Au nanostructures composed of solid solutions (**Figure** **1**d‐I). The average at% of Cu and Au, evaluated by high‐angle annular dark‐field scanning transmission electron microscopy (HAADF‐STEM) with energy‐dispersive spectroscopy (EDS) mapping, were 79% and 21%, respectively, that is, the binary Cu_0.79_Au_0.21_ (Figure 1h). The binary nanostructures appear not to show a helicity at the nanoscale due to small *P* (inset in Figure 1a‐I; Figure S4a‐I,b‐I, Supporting Information), and are polycrystalline with dominant crystal orientation directions along Cu〈111〉 and Au〈111〉 (Figures S4d‐I and S5a, Supporting Information).

The Cu:Au nanostructures exhibit localized surface plasmon resonance (LSPR) with the transverse mode at ≈583 nm (Figure 1a‐I). Circular dichroism (CD) has been widely used to characterize the optical activity of a chiral matter, denoting the differential absorption/extinction of LH and RH circularly polarized light. On resonance with the transverse LSPR, the binary Cu:Au nanostructures show the broad bisignate CD signals in the visible/near infrared region (at a wavelength >400 nm, or the chiroplasmonic range, Figure 1b‐I). Changing the chirality of the host Cu CNPs from RH to LH has a negligible effect on the LSPR (or extinction) spectrum (Figure 1a‐I), but causes a flip of their CD signals around the zero‐CD axis (Figure 1b‐I), illustrating that the binary Cu:Au nanostructures are optically active. The CD spectrum of the LH‐binary CNPs appeared not to be mirror image with that of the RH ones with respect to the zero‐CD axis, mainly due to the GLAD mechanic asymmetry of the substrate rotation in clockwise and counterclockwise. We have reported that the chirality can be effectively transferred from the host CNPs to the dopant M using the LbL‐GLAD, due to the diffusion of M into the chiral lattices of the host that is promoted by the GLAD‐induced heating effect.^[^ ^38^ ^]^ The thermally diffused M atoms are spatially located in the chiral fashion duplicated from the chiral lattices of the host CNPs, i.e., the chiral alloying effect. The chiral alloying was observed in the binary Cu:Au nanostructures. Compared to the host Cu CNPs, the binary Cu:Au nanostructures exhibit an additional CD peak at the wavelength of ≈540 nm (marked by yellow arrows, Figure S3d,e, Supporting Information). This CD peak is in well agreement with that monitored in the unary Au CNPs (marked by yellow arrow, Figure S3f, Supporting Information). Note that the agreement lies in not only the wavelength location of the peak, but also the sign: the RH/LH Au CNPs display the positive/negative CD signals at ≈520 nm, respectively, in coincident with that the Au alloying in the RH/LH Cu CNPs gives rise to the positive/negative CD signals at ≈540 nm, respectively. It is clearly illuminated that the dopant Au shows the chirality duplicated from the host Cu CNPs. Hence, the chiral alloying effect is confirmed in the binary Cu:Au nanostructures, that is, the formation of the binary RH/LH‐CNPs composed of the RH/LH chiral lattices of both the host Cu and dopant Au, respectively. Note that the chiral alloying of the host with dopant Au causes the red shift (i.e., from 520 to 540 nm) in the CD signals of dopant Au, owing to the change in the refractive index of the medium surrounding the dopants.^[^ ^12^ ^]^

To quantitatively evaluate the average optical activity of individual CNPs in the close‐packed arrays deposited by the LbL‐GLAD, the CD signals of a CNP array was normalized by its extinction (in terms of the CNP density and height *H* in the close‐packed array) to evaluate the anisotropy *g*‐factor, according to (Section S1, Supporting Information)

(1) $$\begin{array}{c} g=\mathrm{CD}/\left(16500\;\mathrm{Extinction}\right) \end{array}$$

where CD is the ellipticity (units: millidegree, or mdeg). The CNPs containing high density of the atomically chiral lattices will have large anisotropy *g*‐factor. The binary Cu_0.79_Au_0.21_ CNPs exhibit an anisotropy *g*‐factor of 0.005–0.045 in the chiroplasmonic wavelength range (Figure 1c‐I).

### 2.2 Ternary Cu:Au:Pt CNPs

At room temperature the CSTs made of the binary Cu_0.79_Au_0.21_ CNPs were treated with the GRR in 10 µmol L^−1^ K_2_PtCl_4_, which was continuously stirred at a rate of 380 rpm for as long as 9 h. According to electrode potential (Table S1, Supporting Information), the host Cu will be oxidized and dissolved in the electrolyte, and the simultaneous reduction of PtCl_4_^2−^ anions will lead to the precipitation of Pt on the binary CNPs. Au has an electrode potential (+0.93 V) higher than that of PtCl_4_^2−^/Pt (+0.76 V), so that the chiral dopant Au will not be oxidized during the GRR. The GRR‐mediated replacement of Cu with Pt was verified by the X‐ray photoelectron spectroscopy (XPS) characterization, where the XPS signals of Cu 2p decreased in their amplitude (Figure 1e) and those of Pt 4f rose (Figure 1g) with elongating the GRR. The characterization of HAADF‐STEM with EDS mapping showed that the 5 and 9 h GRR resulted in the generation of the ternary Cu_0.85_Au_0.13_Pt_0.02_ and Cu_0.23_Au_0.56_Pt_0.21_ (Figure 1h). It is interesting to find that the 9 h GRR causes an increase of the at% of Au in the ternary nanoproducts compared to that of the CSTs (Figure 1f, h). One Cu atom could be oxidized to either one Cu^+^ or Cu^2+^ cation in the GRR, through donating either one or two electrons, respectively (Table S1, Supporting Information). If the host Cu atoms were oxidized to Cu^+^ cations, the precipitation of one Pt atom (resulting from accepting two electrons) would lead to the dissolution of two Cu atoms, so that the nonreacted Au would increase in its atomic percent in the ternary nanoproducts. By contrast, if Cu was oxidized to Cu^2+^ cations, the dissolution of one Cu atom would be accompanied with the precipitation of a Pt atom, and thus there would be no change in the at% of Au. Consequently, it can be derived that in the GRR of the Cu:Au CNPs, the host Cu is oxidized to Cu^+^ cations (Table S1, Supporting Information). We also found that the 5 h GRR made the at% of Cu rise from 79% in the CSTs to 85% in the ternary nanoproducts, apparently not in coincide with the GRR‐mediated dissolution of the host Cu. We will discuss this phenomenon in Section 2.5.

The replacement of Cu with Pt tends to initially occur at the surfaces of the binary CNPs, and Au mainly exists in the cores (Figure 1d‐II). Then the replacement gradually happens from the external to the internal portions (Figure 1d‐III). Cu and Pt atoms appear to form the solid solutions, and there is segregation of Au atoms in the Cu_0.23_Au_0.56_Pt_0.21_ nanoparticles. The ternary nanoproducts appear to be partially mesoporous (insets in Figure 1a‐II,III; Figure S4b‐II,III, Supporting Information) and tend to slightly increase in their diameters with the evolution of GRR (Figure S4c, Supporting Information). The ternary nanoparticles are polycrystalline with dominant crystal orientation directions along 〈111〉 of the three elements (Figure 1i‐III; Figures S4d‐II,III and S5b,c, Supporting Information).

Importantly, the GRR nanoproducts show the CD signals flipping around the zero‐CD axis with switching the helicity of the binary CSTs from the LH to RH (Figure 1b‐II,III), illustrating that the nanoproducts are CNPs inherently having optical activity. Compared to the CSTs, the ternary CNPs show an additional LSPR peak at the wavelength of ≈360 nm and a CD peak on resonance with the additional LSPR peak (marked by green background, Figure 1a‐II,III,b‐II,III). The resonant CD peak has positive/negative sign at the wavelengths of 300–500 nm as a result of the GRR of the LH/RH‐CSTs, respectively. The emerging LSPR and CD signals are proposed to be assigned to the precipitating Pt. To verify this hypothesis, unary Pt CNPs were deposited by GLAD‐FSR showing broad CD signals at the wavelengths of 200–800 nm (Figure S6, Supporting Information). The LH/RH Pt CNPs exhibit positive/negative CD signals at the wavelengths of 300–500 nm, respectively, in good agreement with the CD signals of the ternary Cu:Au:Pt CNPs. It illustrates that the GRR‐induced emerging CD peak is assigned to Pt, and the precipitating Pt atoms tend to duplicate the chirality of the CSTs, that is, the GRR of the LH/RH‐binary Cu:Au CNPs enables the formation of the LH/RH‐ternary Cu:Au:Pt CNPs in which the three elements all exhibit the LH/RH‐chirality, respectively. In the other word, the GRR can effectively mediate the chirality transfer from the binary to ternary CNPs, which has also been observed from the unary to binary CNPs.^[^ ^35^ ^]^ The Pt‐related CD peaks in the ternary CNPs appear to have blue shift compared to the unary Pt CNPs, probably owing to the ternary alloying in the CNPs. The elongation of GRR from 5 to 9 h leads to the amplification of the anisotropic *g*‐factor of Pt and simultaneously weakens the bisignate optical activity of the binary metals at the wavelengths of 500–800 nm (Figure 1c(I–III)), as a result from the chiral replacement of Cu with Pt. When the binary CSTs and ternary CNPs were dispersed in water, there was only one CD peak detected at the wavelengths of 500–800 nm (Figure S7, Supporting Information). Different from the dispersed CNPs, in the close‐packed arrays of the vertically protruding CNPs there is the chiroplasmonic coupling of neighboring CNPs and the CD spectrum was monitored at the incident along the growth axes of the protruding CNPs. Therefore, it can be derived that the bisignate Cu:Au CD peaks originate from the chiroplasmonic coupling and/or the anisotropic growth orientation of the CNPs.

Note that the current nanocharacterization technique could not provide the elemental mapping at the atomic resolution. Given that the structural chirality of the CNPs originates from the atomically chiral lattices, it is very challenging to disclose the GRR‐mediated evolution of the chirality transfer in the binary CNPs at the atomic scale.

### 2.3 Ternary Cu:Au:Ag CNPs

**Figure 2:**
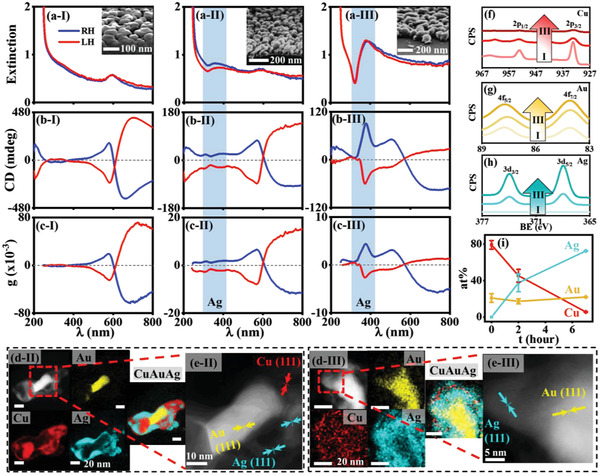
Generation of ternary Cu:Au:Ag CNPs, through the GRR of binary Cu:Au CNPs in an aqueous electrolyte containing 20 µmol L^−1^ AgNO_3_, as a function of *t*: (I) 0, (II) 2, and (III) 7 h. In the CST binary CNPs, the nominal *P* of the host Cu is ≈10 nm and *T*_Au_ is 20 nm. The GRR was performed at 30 °C through stirring the electrolyte at a rate of 380 rpm, to generate ternary Cu:Au:Ag CNPs. UV/visible spectra: a) extinction, b) CD, c) anisotropic *g*‐factor. (a–c) Blue lines: the GRR of RH‐Cu:Au CNPs; red lines: the GRR of LH‐Cu:Au CNPs. Sky blue backgrounds are used to mark the chiroplasmonic signals of the precipitating Ag. Insets: (a‐I, a‐II, a‐III) SEM oblique images of the samples. d) HAADF‐STEM images (in black and white) and the corresponding EDS at% mapping of Cu (in red), Au (in yellow), Ag (in sky blue), and their mixtures, as a function of *t* (scale bar: 20 nm). e) HRSTEM images of the portions of the ternary CNPs marked by red dash squares in (d). XPS spectra of the binary CNPs treated with the GRR as a function of *t*: f) Cu 2p, g) Au 4f, h) Ag 3d. (f–h) Gradually darkening colors represent an elongation of the GRR from (I) 0 h to (III) 7 h. i) Plot of the mean at% of Cu, Au, and Ag in the CNPs versus *t*, evaluated from the EDS at% mapping of more than five individual CNPs. Error bars represent SD. Insets in (a‐I, II, III) and (d–i): the GRR of the RH‐binary CNPs.

The chirality transmission from the binary to ternary CNPs was also observed in the GRR of the Cu_0.79_Au_0.21_ CNPs in 20 µmol L^−1^ AgNO_3_. It was at the AgNO_3_ concentration of 20 µmol L^−1^ that the chirality transfer from Cu to Ag was maximized.^[^ ^35^ ^]^ The oxidation and dissolution of the host Cu, accompanied with the reduction of Ag^+^ cations and the resultant precipitation of Ag on the binary CNPs (Table S1, Supporting Information), lead to the generation of the ternary Cu:Au:Ag CNPs (**Figure** **2**). Au has an electrode potential (+0.93 V) higher than that of Ag (+0.80 V), so that the chiral dopant Au is resistant to the galvanic oxidation and dissolution. The GRR lasting for 2 and 7 h resulted in the formation of Cu_0.45_Au_0.17_Ag_0.38_ and Cu_0.06_Au_0.22_Ag_0.72_ CNPs (Figure 2i), respectively, which were optically active (Figure 2a–c,I–III). The ternary CNPs were polycrystalline with dominant crystal orientation directions along 〈111〉 of the three elements (Figure 2e‐II,III; Figure S8d, Supporting Information). The replacement of Cu with Ag starts to take place at the surfaces of the binary CNPs, and Au mainly exists in the cores (Figure 2d‐II). Then the replacement gradually happens from the external to the internal portions, and there is segregation of Ag and Au in the ternary CNPs (Figure 2d‐III). The GRR gives rise to a decrease of the Cu at% and increase of the Ag at%, and notably the Au at% has no obvious change with the GRR (Figure 2f–i). It further verifies the anodic oxidation of the host Cu to Cu^+^ cations, so that the dissolution of a Cu atom results in transferring one electron to reduce one Ag^+^ cation and thus causes the precipitation of one Ag atom. It accounts for no change in the at% of the inert Au in the CNPs.

Additional LSPR and the resonant CD signals at the wavelength of ≈390 nm were monitored in the GRR evolution (marked by sky blue background, Figure 2a‐II,b‐II,a‐III,b‐III), assigned to the transverse chiroplasmonic mode of the precipitating Ag.^[^ ^23^ ^]^ The GRR of the LH/RH Cu_0.79_Au_0.21_ CNPs caused the emerging transverse CD mode of Ag to have negative/positive sign, respectively. The LH/RH unary Ag CNPs, deposited by GLAD‐FSR, show the transverse CD mode at the wavelength of ≈370 nm with negative/positive sign, respectively (marked by sky blue background, Figure S9, Supporting Information). It is strongly illustrated the GRR‐mediated chirality duplication from the binary CSTs to Ag, that is, the GRR of the LH/RH binary CNPs produces the LH/RH Ag in the ternary CNPs, respectively. It is the chiral alloying that gives rise to the formation of the ternary Cu:Au:Ag CNPs where the three elements exhibit the same chirality at the atomic scale. Compared to the unary Ag CNPs, the Cu_0.06_Au_0.22_Ag_0.72_ CNPs, as a result from the 7 h GRR, exhibit the broader transverse CD mode of Ag with a red shift. The chiral alloying of the binary Cu:Au CNPs with Ag causes the change in the refractive index of the Ag's medium, accounting for the CD red shift. The GRR made the CNPs rise in their diameter from 62 ± 14 nm of the binary CNPs to 152 ± 48 nm (represented in average value ± standard deviation, Figure S8a–c, Supporting Information). The GRR‐induced increase in the diameter distribution (i.e., the standard deviation of 48 nm vs 14 nm) results in broadening the CD profile. The ternary Cu:Au:Ag CNPs are less optically active than their CSTs (Figure 2c‐III and c‐II vs Figure 2c‐I), probably ascribed to the relaxation of the metastable chiral lattices to thermodynamically stable achiral structures stimulated by chemical energy released by the GRR, as well as to the incomplete chirality transmission through the GRR.^[^ ^35^ ^]^

Although the GRR of the unary Cu CNPs in the K_2_PtCl_4_ or HAuCl_4_ electrolyte could not enable the formation of the binary Cu:Pt (Figure S1, Supporting Information) or Cu:Au (Figure S2, Supporting Information) CNPs through chirality transfer, respectively, the chiral doping of the Cu CNPs with Au successfully led to the GRR‐mediated chirality transmission (Figure 1; Figure S10, Supporting Information). Hence, it is the doped Au that plays a vital role in the GRR‐mediated chirality transmission from the Cu CNPs. Au with relatively high electrode potential is resistant to the anodic oxidation/dissolution in the given electrolytes, and thus we hypothesize that the dopant Au serves as a structural scaffold to assist the GRR‐mediated chirality transfer.

### 2.4 Functions of the Dopant Au in the CSTs

To verify the hypothesized function of the dopant Au, alternatively Ag was doped in the host Cu CNPs to generate the binary Cu:Ag CNPs (Figure S11a‐I,b‐I, Supporting Information). In a 10 µmol L^−1^ K_2_PtCl_4_ electrolyte, the GRR of the binary Cu:Ag CNPs could not mediate the chirality transfer to generate the ternary Cu:Ag:Pt CNPs (Figure S11, Supporting Information). Ag has a relatively low electrode potential (+0.22 V, Table S1, Supporting Information) in an electrolyte containing Cl^−^ anions, so that the dopant Ag will be oxidized and dissolved in the electrolyte. Without the scaffold of the dopant Ag, the GRR could not effectively mediate the chirality transfer from the host Cu CNPs.

**Figure 3:**
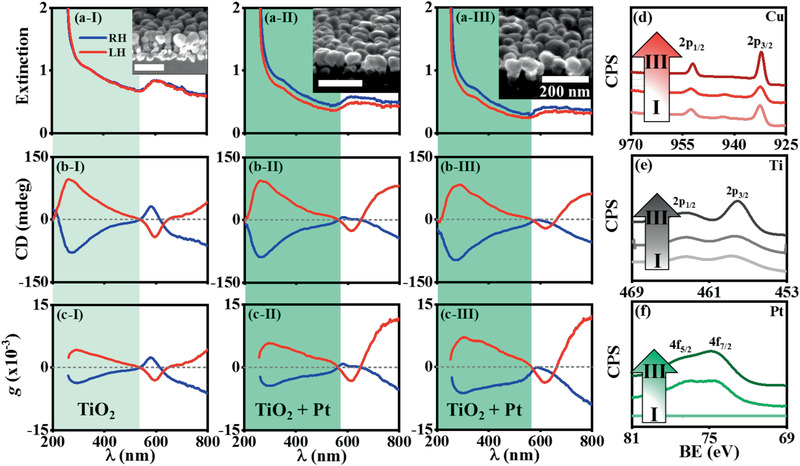
Production of Cu:TiO_2_:Pt CNPs, through the GRR of Cu:TiO_2_ CNPs in an aqueous electrolyte containing 10 µmol L^−1^ K_2_PtCl_4_, as a function of *t*: (I) 0, (II) 5, (III) 9 h. *P* of the host Cu is ≈10 nm, and the nominal thickness of TiO_2_ is 20 nm. The GRR was performed at room temperature through stirring the electrolyte at a rate of 380 rpm, to generate Cu:TiO_2_:Pt CNPs. UV/visible spectra: a) extinction, b) CD, c) anisotropic *g*‐factor. (a–c) Blue lines: the GRR of RH‐Cu:TiO_2_ CNPs; red lines: the GRR of LH‐Cu:TiO_2_ CNPs. (a–c): (I) Light green background is used to mark the chiroplasmonic signals of TiO_2_; (II, III) green background is used to mark the chiroplasmonic signals of TiO_2_ and the precipitating Pt. Insets: (a‐I, a‐II, a‐III) SEM oblique images of the samples. XPS spectra of the Cu:TiO_2_ CNPs treated by the GRR as a function of *t*: d) Cu 2p, e) Ti 2p, f) Pt 4f. (d–f) Gradually darkening colors represent an elongation of the GRR from (I) 0 h to (III) 9 h. Insets in (a‐I, II, III) and (d–f): the GRR of the RH‐Cu:TiO_2_ CNPs.

Furthermore, Au was replaced with TiO_2_ being doped in the host Cu CNPs (**Figure** **3**a‐I,b‐I,c‐I). TiO_2_ has an electrode potential of +0.762 V in an acidic electrolyte (Table S1, Supporting Information), and thus is also resistant to the oxidation in the K_2_PtCl_4_ electrolyte. TiO_2_ can function as the structural scaffold and thus lead to the formation of the Cu:TiO_2_:Pt CNPs through the GRR (Figure 3a–c(II–III),d–f). The TiO_2_ CNPs, deposited by GLAD‐FSR, show the chiroptical region in the wavelength range of 200–400 nm due to electronic transitions under asymmetric electric fields,^[^ ^39^ ^]^ and the CD signals are positive/negative for the LH/RH chirality, respectively (Figure S12a,b, Supporting Information). Alloying the host Cu CNPs with TiO_2_ makes the TiO_2_‐related chiroptical region extend to 200–520 nm (highlighted by light green background, Figure 3a–c(I)). In this chiroptical region, doping TiO_2_ in the LH/RH‐Cu CNPs leads to the CD signals with positive/negative sign, illuminating that TiO_2_ is induced to have the LH/RH chirality, respectively, i.e., the chiral alloying of the CSTs with TiO_2_. Note that the chiroptical region of the chirally doped TiO_2_ overlaps with that of Pt but does not with that of Cu (Figure 1c‐II,III). The galvanic replacement of Cu with Pt, through the GRR of the Cu:TiO_2_ CNPs, causes the gradual increase in the average optical activity (evaluated by the anisotropic *g*‐factor) of individual nanoproducts with elongating the GRR at the wavelengths <580 nm (Figure 3c‐II,III; Figure S12c, highlighted by green background, Supporting Information), because the precipitating Pt duplicated with the LH/RH chirality of the CSTs also show the positive/negative CD signals, respectively.

These controlled experiments clearly verify that the electrochemically inert materials (such as Au) function as the structural scaffold to assist the GRR‐mediated chirality transfer from the host Cu CNPs to another metal, resulting in the formation of the ternary CNPs.

### 2.5 Kinetics of the Au‐Assisted Chirality Transfer

**Figure 4:**
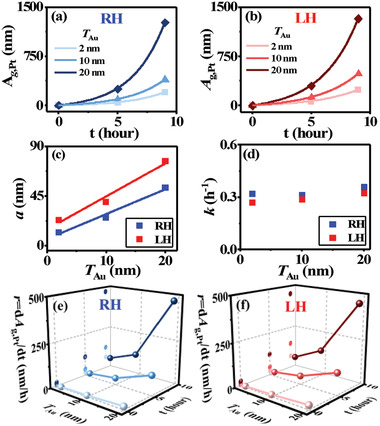
Kinetics of the Au‐assisted chirality transfer. The GRR of binary Cu:Au CNPs in an aqueous electrolyte containing 10 µmol L^−1^ K_2_PtCl_4_, as a function of *t*: (I) 0, (II) 5 h, and (III) 9 h. *T*_Au_ in the binary CNPs is 2, 10, 20 nm. The GRR was performed at room temperature, through stirring the electrolyte at a rate of 380 rpm. Plots of *A_g_*_,Pt_ (denoting the integrated area of the anisotropic *g*‐factor peak associated with the precipitating chiral Pt) versus *t*, as a function of *T*_Au_: a) the GRR of RH‐Cu:Au CNPs, b) the GRR of LH‐Cu:Au CNPs. c) Plots of the prefactor *a* versus *T*_Au,_ which are linearly fitted. d) Plots of the reaction rate coefficient *k* versus *T*_Au_. (c,d) Blue squares: the GRR of RH‐Cu:Au CNPs; red squares: the GRR of LH‐Cu:Au CNPs. e,f) Plots of the chirality transfer rate *r* versus *T*_Au_ and *t*: (e) the GRR of RH‐Cu:Au CNPs and (f) the GRR of LH‐Cu:Au CNPs.

It is of fundamental importance to study the kinetics of the Au‐assisted chirality transfer. *T*_Au_ was tailored to be 2 (Figure S13, Supporting Information), 10 (Figure S14, Supporting Information), and 20 nm (Figure 1) in the binary CSTs, which were treated with the GRR in 10 µmol L^−1^ K_2_PtCl_4_ at room temperature. Only a portion of the precipitating Pt duplicate the chiral lattices of the CSTs, so that it is not appropriate to study the chirality transfer kinetics through evaluating the GRR‐mediated change in the Pt composition. Instead, the integrated area of the anisotropic *g*‐factor peak assigned to the precipitating chiral Pt (*A_g_*_,Pt_, in units of nm) was measured (e.g., inset in Figure S13c‐II,c‐III, Supporting Information) as a function of the helical handedness of the binary CSTs, *T*_Au_, and *t*, summarized in **Figure** **4**a,b. Although the binary CSTs showed the dielectric *g*‐factor peak in the wavelength region of 250–400 nm (Figure 1c‐I; Figures S13c‐I and S14c‐I, Supporting Information) partially overlapping with that of the precipitating Pt in the ternary CNPs, the integrated area of the dielectric *g*‐factor peak is negligible and thus it will not affect the kinetic evaluation. The kinetics of the Au‐assisted chirality transmission from the binary Cu:Au CNPs to the precipitating Pt was quantitatively evaluated with monitoring *A_g_*_,Pt_ as a function of *t* in the range of 0–9 h, given that *A_g_*_,Pt_ is proportional to the amount of the precipitating chiral Pt. The plots of *A_g_*_,Pt_ versus *t* (Figure 4a,b) were well fitted with

(2) $$\begin{array}{c} A_{g,\mathrm{Pt}}=a\left(e^{\mathrm{kt}}-1\right) \end{array}$$

where *k* is the reaction rate coefficient (in units of h^−1^), and *a* is the prefactor (in units of nm). At *t* = 0 h, there is no chirality transfer and thus *A_g_*_,Pt_ = 0. Equation (2) illustrates that the Au‐assisted chirality transfer from the Cu:Au CNPs is the first‐order reaction. The fitting shows that the prefactor *a* tends to linearly rise with an increase of *T*_Au_ at a slope of 2.2 and 3.0 for the GRR of the RH‐ and LH‐CSTs, respectively (Figure 4c). By contrast, *k* has not obvious change with *T*_Au_, with an average value of 0.33 and 0.29 h^−1^ at room temperature for the GRR of the RH‐ and LH‐CSTs, respectively (Figure 4d). In principle, the GRR should follow the first‐order kinetics independent on the chirality of the CSTs. Note that GLAD‐FSR enables the deposition of the close‐packed arrays of the binary CNPs with wide distribution of the helical structures and uncontrollable structural defects, probably accounting for the observed difference in *a* and *k* for the GRR of the LH‐ and RH‐Cu:Au CNPs.

The chirality transfer rate (*r*) was calculated by differentiating *A_g_*_,Pt_ with respect to *t* and summarized in Figure 4e,f, in terms of the GRR of the RH‐ and LH‐binary CNPs, respectively. *r* generally increases with thickening the sandwiched Au layers at a given *t*. At *t* of 5 h, for example, *r* for the GRR of the RH‐binary CNPs increases from 19.1 nmh^−1^ at *T*_Au_ = 2 nm to 37.7 nm h^−1^ at *T*_Au_ = 10 nm and 112.7 nm h^−1^ at *T*_Au_ = 20 nm; and the GRR of the LH‐CSTs shows an increase of *r* from 24.3 nm h^−1^ at *T*_Au_ = 2 nm to 47.9 nm h^−1^ at *T*_Au_ = 10 nm and 124.6 nm h at *T*_Au_ = 20 nm. The CSTs of the binary Cu:Au CNPs are composed of the solid solution (Figure 1d). The larger the *T*_Au_, the more Au chirally dissolved in the solvent Cu. More structural scaffolds of the chiral Au lattices in the host Cu CNPs is favored for the GRR‐mediated chirality transfer, resulting in larger *r*.

**Scheme 1:**
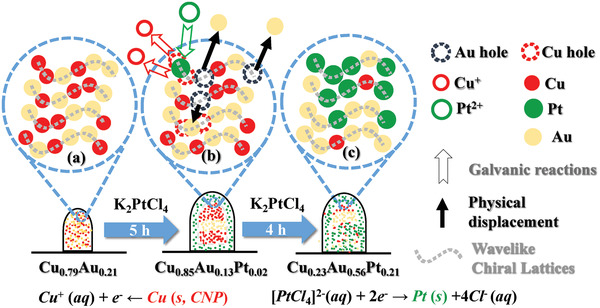
Schematic illustration of the GRR‐mediated chirality transfer from a) the binary Cu_0.79_Au_0.21_ CSTs to b,c) the ternary Cu:Au:Pt CNPs, as a function of GRR duration.

The evolution of the Au‐assisted GRR is proposed in **Scheme** **1**. Due to lacking the atomic resolution of the elemental mapping, the chiral lattice patterns are unknown. Here, the wavelike chiral lattices^[^ ^30^ ^]^ are used to present the chiral lattices in the binary CSTs (Scheme 1a) and the ternary products (Scheme 1b,c). Initially, the chiral Cu lattices in the Cu:Au solid solutions are replaced with metal M (Pt and Ag), which occurs in the external portions of the CSTs (Scheme 1b). Meanwhile, the Au chiral lattices in the template solid solutions are galvanically resistant to the GRR. The precipitating M atoms tend to aggregate in a chiral pattern duplicating that of the Au chiral lattices, and thus the Au chiral lattices function as the chiral scaffold to support and guide the GRR‐mediated chirality transmission. Concurrently, the inert Au chiral lattices tend to aggregate in the cores by occupying the interior holes generated by the galvanic dissolution of Cu atoms and some Au peels off from the CSTs (Scheme 1b), accounting for the decrease of the Au at% from 21% in the CSTs to 13% after the 5 h GRR in 10 µmol L^−1^ K_2_PtCl_4_ (Figure 1h) and to 17% after the 2 h GRR in 20 µmol L^−1^ AgNO_3_ (Figure 2i). The 5 h GRR of the Cu_0.79_Au_0.21_ CNPs only causes the precipitation of 2 at% Pt (Figure 1h) so that the loss of Au is superior to the Cu dissolution, resulting in an increase of the Cu at% from 79% in the CSTs to 85% in the ternary Cu:Au:Pt CNPs. By contrast, the 2 h GRR gives rise to the precipitation of 38 at% Ag; hence, the Cu oxidation is superior to the loss of Au, accounting for the decreases of the Cu at% to 45% in the ternary Cu:Au:Ag CNPs. Then the replacement of Cu with metal M gradually takes place from the external to the internal portions (Scheme 1c), to form the ternary CNPs with phase segregation.

### 2.6 Ambient Aging of the Ternary CNPs

**Figure 5:**
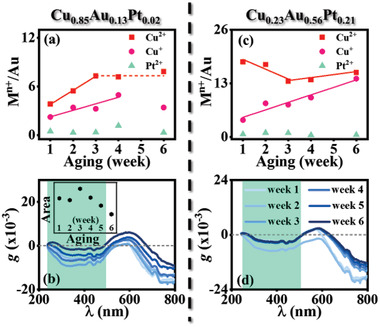
Ambient aging of the ternary RH‐CNPs. a,b) Cu_0.85_Au_0.13_Pt_0.02_ (Figure 1b‐II). c,d) Cu_0.23_Au_0.56_Pt_0.21_ CNPs (Figure 1b‐III). (a,c) Plots of the at% ratio of M*^n^* ^+^/Au (M*^n^* ^+^: Cu^+^, Cu^2+^, and Pt^2+^) as a function of aging duration. Solid lines represent the linear fitting within different aging durations. (b,d) UV/visible Anisotropic *g*‐factor spectra of the ternary CNPs as a function of aging duration. The broad Pt‐related peaks are highlighted with green background. Inset: (b) the integrated area of the broad Pt‐related peaks as a function of aging duration.

Chiroptical stability of the ternary CNPs is of fundamental interest for the exploration of potential applications in, for instance, asymmetric catalysis. The ternary Cu:Au:Pt CNPs were selected to study, because the three compositional metals widely function as catalysts. Spontaneous oxidation of the Cu:Au:Pt CNPs in the ambient conditions was monitored as a function of aging duration within 6 weeks. Characterized with XPS, the ternary surfaces contain Cu^+^, Cu^2+^, and Pt^2+^ cations due to the spontaneous oxidation and Au is inert to the oxidation (Figures S15d–f and S16d–f, Supporting Information). To quantitatively evaluate the surface oxidation, the at% ratio of the metallic cations (M*^n^* ^+^: Cu^+^, Cu^2+^, and Pt^2+^) to inert Au atoms was evaluated as a function of aging duration. For the Cu_0.85_Au_0.13_Pt_0.02_ CNPs, the at% ratio of Cu^2+^/Au linearly rises within the 3‐week aging at a slope of 1.7 per week, and then reaches at a plateau of 7.3 from 3 to 6 weeks (**Figure** **5**a). Differently, the at% ratio of Cu^+^/Au has a linear increase within the 4‐week aging at a slope of 0.8 per week, followed by a slight decrease in the 6th week. The at% ratio of Pt^2+^/Au barely varies within the 6‐week aging, in spite of a slight increase in the 4th week. It is illustrated that at the ternary CNP surfaces Cu is oxidized to Cu^2+^ faster than to Cu^+^ in the first 3 weeks (because the electrode potential $E_{Cu^{2+}/\mathrm{Cu}}<E_{Cu^{+}/\mathrm{Cu}}$, Table S1, Supporting Information) and then the Cu oxidation is prohibited, and Pt has good resistance to the ambient oxidation due to its relatively high electrode potential (Table S1, Supporting Information). For the Cu_0.23_Au_0.56_Pt_0.21_ CNPs, the at% ratio of Cu^+^/Au linearly rises within the 6‐week aging at a slope of 3.0 per week and the antioxidation of Pt was also observed (Figure 5c). Surprisingly, the at% ratio of Cu^2+^/Au tends to linearly reduce within the 3‐week aging at a slope of −2.4 per week, followed by a linear increase from 3 to 6 weeks at a slope of 2.1 per week. The reason is still ambiguous. Compared to the Cu_0.85_Au_0.13_Pt_0.02_ CNPs, the Cu_0.23_Au_0.56_Pt_0.21_ CNPs have the faster oxidation of Cu to Cu^+^ at the CNP surfaces.

Furthermore, the optical properties of the ternary Cu:Au:Pt CNPs were monitored to show that the ambient aging has a negligible effect on the extinction (Figures S15a and S16a, Supporting Information) but obviously affect the chiroptical activity in terms of the anisotropic *g*‐factor (Figure 5b,d). For the Cu_0.85_Au_0.13_Pt_0.02_ CNPs, the Pt‐related optical activity (highlighted with green background in Figure 5b) tends to be amplified in the first 3 weeks and then quickly suppressed with the aging in the next three weeks (inset in Figure 5b). Considering the antioxidation of Pt and the oxidation of Cu, this broad peak can be assigned to the Cu:Pt alloy. Moreover, the bisignate peaks (at the wavelength >500 nm) assigned to the binary host Cu:Au CNPs tend to have an upshift with the aging. By contrast, the Cu_0.23_Au_0.56_Pt_0.21_ CNPs show the stabilization of the optical activity after the 3‐week aging (Figure 5d). High composition of the inert elements (Au and Pt) is favored for the ternary Cu:Au:Pt CNPs to have the antiaging optical activity. The comparison (Figure 5a vs 5b, and Figure 5c vs 5d) illuminates the decoupling of the compositional oxidation and the chiroptical evolution.

The Cu_0.23_Au_0.56_Pt_0.21_ CNPs possess the aging‐stabilized optical activity, while there are Pt(II) and the continuous oxidation of Cu to Cu(I) at the surfaces. It is indicated that the Cu_0.23_Au_0.56_Pt_0.21_ CNPs promisingly function as the asymmetric catalyst. On the one hand, the ternary CNPs consist of the atomically chiral lattices at the surfaces where molecular substrates will enantiospecifically adsorb,^[^ ^27^ ^]^ which is the prerequisite to induce the asymmetric synthesis. The aging‐stabilized optical activity results from the preservation of the chiral lattices with the aging, which is indispensable to maintain the asymmetric catalytic activity. On the other hand, it has been well known that Cu (I) and Pt (II) play a catalytic role in, for example, the enantioselective arylation of benzylic radicals^[^ ^40^ ^]^ and the asymmetric aldol and silylcyanation reactions,^[^ ^41^ ^]^ respectively. The ternary Cu_0.23_Au_0.56_Pt_0.21_ CNPs will serve as the asymmetric catalyst with no assistance of chiral ligands.

## 3 Conclusion

In conclusion, a thin layer of galvanically “inert” dopants, such as Au, is doped in the host Cu CNPs using LbL‐GLAD to generate the binary Cu:Au CNPs in which the dopants diffuse into the host to form the solid solutions and duplicate the atomic chirality of the host, due to the heating‐induced chiral alloying effect. The “inert” dopants serve as the structural scaffold to assist the chirality transmission from the host Cu CNPs to the third metals (M: Pt and Ag) cathodically precipitating on the CNPs, leading to the formation of the ternary Cu:Au:M CNPs where the three constituent metals show the same atomic chirality as the host Cu CNPs. The ternary CNPs are polycrystalline with dominant crystal orientation directions along 〈111〉 of the three components, and their compositions are tailored with engineering the GRR duration. In the evolution of the Au‐assisted GRR of the binary Cu:Au CNPs, the precipitating M starts to galvanically replace the host Cu at the surfaces of the binary CNPs and the dopant Au tends to aggregate in the cores, followed by the continuous replacement of the host Cu with M in the internal portions of the CNPs. There is phase segregation in the ternary CNPs. More scaffold Au dopants are favored for the faster chirality transfer through the GRR. The Au‐assisted chirality transfer from the host Cu CNPs follows the first‐order kinetics with the *k* of ≈0.3 h^−1^ at room temperature. The Cu_0.23_Au_0.56_Pt_0.21_ CNPs contain Cu(I) and Pt(II) at the surfaces and have the aging‐stabilized optical activity, promisingly functioning as the asymmetric catalysts. This work enables further understanding of the GRR‐mediated chirality transfer, and provides the extension of compositional space from the binary to ternary of alloy CNPs that may pave the way toward enhancing the functions of prominent chirality‐associated applications in the fields of enantiodifferentiation, enantioseperation, asymmetric catalysis, bioimaging, and biodetection.

## 4 Experimental Section

##### GLAD‐FSR of Unary CNPs

The unary CNPs were made from Cu, Ag, and Pt. In a custom‐built physical vapor deposition system (JunSun Tech Co. Ltd., Taiwan) with a high vacuum of 10^−7^–10^−6^ Torr, metallic pellets (Cu: 99.999%, Fuzhou Innovation Photoelectric Technology Co., Ltd., China; Ag, Pt: 99.99%, Kurt J. Lesker) were evaporated at a deposition angle (*α*) of 86° with respect to the direction normal to the substrate, using an electron‐beam accelerating voltage of 8.0 kV. Cu, Ag, and Pt were evaporated at a rate of ≈0.3, 0.3, and 0.05 nm s^−1^ as monitored by a quartz crystal microbalance that was located near the substrate, using emission current of 40–50, 15–25, and 120–130 mA, respectively. The substrate temperature (*T*_sub_) was set at ≈−5 °C for Cu and −45 °C for Ag and Pt during the deposition, using an ethanol cooling system. To produce the RH/LH CNPs with a given nominal helical pitch (*P*), a substrate was rotated clockwise/counterclockwise, respectively, at a rate *R*_r_ (in units of degree per second, or ° s^−1^) given by

(3) $$\begin{array}{c} R_{r}=360\;R_{d}/P_{d} \end{array}$$

where *R*_d_ is the deposition rate of the metals at the substrate surface calibrated at *α* of 86°. *R*_d_ was calibrated as 0.078, 0.045, and 0.010 nm s^−1^ for Cu, Ag, and Pt, respectively. *P*_d_ is the as‐designed nominal *P*, which was 10 nm for Cu, Ag, and Pt. The nominal *P* was experimentally evaluated by

(4) $$\begin{array}{c} P=H/m \end{array}$$

where *H* is the helical height that was measured by scanning electron microscopy (SEM, Oxford, LEO 1530), and *m* is the number of substrate rotations during GLAD. It was found out that the measured *P* was in well agreement with *P*_d_.^[^ ^23^, ^28^ ^]^ The CNPs were also made from TiO_2_. At *α* of 86° and *T*_sub_ of 5 °C, TiO_2_ pellets (99.9%, Kurt J. Lesker) were evaporated at *R*_d_ of 0.040 nm s^−1^, using an electron‐beam accelerating voltage of 8.0 kV and emission current of 59 mA. The TiO_2_ CNPs had a *P* of ≈10 nm. The samples were deposited on Si wafer (Semiconductor Wafer Inc., Taiwan) and sapphire (Meco Technology Ltd., Hong Kong) over an area of 1.5 × 1.5 cm^2^.

##### LbL‐GLAD of Binary Cu:M CNPs (M: Au, Ag, TiO_2_)

Binary CNPs were generated through three‐step LbL‐GLAD processes.^[^ ^38^ ^]^ The first and third step was the GLAD‐FSR of the host unary Cu CNPs having a nominal *P* of roughly 10 nm and *H* of ≈50 nm, and the second step was the GLAD of dopant M without substrate rotation, with a nominal thickness of *T*_M_. M included Au (99.999%, Fuzhou Innovation Photoelectric Technology Co., LTD), Ag (99.99%, Kurt J. Lesker), and TiO_2_ (99.9%, Kurt J. Lesker). The second GLAD process was performed at *α* of 86°, *T*_sub_ ≈ −5 °C and *R*_d_ = 0.1 nm s^−1^, using an electron‐beam accelerating voltage of 8.0 kV and emission current of 60 mA for Au, 30 mA for Ag, and 40 mA for TiO_2_. There was a 5 min interval between the two subsequent GLAD processes.

##### GRR of Binary Cu:M CNPs

The electrolyte contained 20 µmol L^−1^ silver nitrate (AgNO_3_, 99.0%, Sigma‐Aldrich), 10 µmol L^−1^ potassium tetrachloroplatinate(II) (K_2_PtCl_4_, 99.99%, Sigma‐Aldrich), and 10 µmol L^−1^ chloroauric acid (HAuCl_4_·3H_2_O, 99.999%, Sigma‐Aldrich). All the GRR were performed at room temperature, except for that of the Cu:Au CNPs in 20 µmol L^−1^ AgNO_3_ at 30 °C. Before the GRR, the Teflon holder and beaker were degreased in Piranha (98% H_2_SO_4_: 30% H_2_O_2_ = 3:1, v/v) for 15 min at room temperature, sufficiently rinsed with DI water (18.2 MΩ, Milli‐Q reference water purification system fed with campus distilled water), and dried with N_2_ gases. After the GRR lasting for a controlled duration, the samples were removed out of the electrolyte to terminate the GRR, followed by thoroughly rinsing with DI water and drying with N_2_ gases.

##### UV/Visible Extinction and CD Spectroscopies

Under ambient conditions, BioLogic CD (MOS 500) was used to monitor UV/visible extinction and CD spectra of the samples deposited on sapphires, under an irradiative incident along the normal direction of the substrates. A sample was continuously rotated clockwise at 0.2 rpm to monitor a CD spectrum in a wavelength range of 200–800 nm, and four CD spectra were subsequently recorded (e.g., Figure S17, Supporting Information). After monitoring each CD spectrum, the sample was manually rotated in an angle of 90° around its normal axis before measuring the next CD spectrum. It was generally observed that the four CD spectra of a given sample could not overlap each other (Figures S17, Supporting Information), owing to the contribution from linear birefringence mainly stemming from the growth orientation of the CNPs on the supporting substrate. Then the four CD spectra were algebraically averaged to obtain a CD spectrum of the sample, to eliminate the linear birefringence.

##### Structure Characterization

The as‐deposited samples were mechanically split, leaving the freshly exposed surfaces for SEM (Oxford, LEO 1530) characterization. SEM top‐down images of the samples were analyzed using ImageJ to statistically evaluate the diameter *D* of the CNPs in an average value ± standard deviation (SD). The CNPs were scratched off the substrates and well dispersed in ethanol through ultrasonication for 5 min. Several drops of the mixture were applied to a lacey carbon film on a grid structure (Electron Microscopy Sciences). The grid was ambiently dried for 1 h and characterized by transmission electron microscopy (TEM, Tecnai G2 20 STWIN). Z‐contrast imaging and EDS mapping of the CNPs was performed by HAADF‐STEM, on a Cs‐corrected Titan Themis 80–300 TEM, operated at 300 kV, fitted with a Bruker Quantax Super‐X EDS detector. Elemental maps were acquired using a probe size of 1–2 nm, with count rates between 800 and 1500 counts per second and acquisition time of 10–15 min. A standard‐based quantification method (Linemarker TEM) was used for elemental quantification of the acquired elemental maps. Multiple individual CNPs (>5) of a given sample were monitored by HAADF‐STEM with EDS to statistically evaluate the mean atomic percentage (at%) ±SD, using Velox 4.0 offline software. The samples were characterized by X‐ray diffraction (XRD, Bruker, nonmonochromated Cu K*α* X‐ray with wavelength of 0.15 418 nm, Advance D8 multipurpose X‐ray diffractometer) and XPS (Sengyang SKL‐12, non‐monochromatic Mg K*α* radiation of 1253.6 eV, at a current of 15 mA, voltage of 10 kV and takeoff angle (between the sample and detector) of 90°, and in a vacuum of ≈2 × 10^−9^ mbar).

## Conflict of Interest

The authors declare no conflict of interest.

## Supporting information

Supporting Information
