# Supplementary material for: Extension of Compositional Space to the Ternary in Alloy Chiral Nanoparticles through Galvanic Replacement Reactions
Source: Adv Sci (Weinh). 2020 Oct 27;7(23):2001321. doi: 10.1002/advs.202001321 (PMC7710001; doi:10.1002/advs.202001321)
Supplement: Supplementary file 1 — Supporting Information [file ADVS-7-2001321-s001.pdf]

WILEY-VCH

© 2020 Wiley-VCH GmbH

ADVANCED SCIENCE Open Access

Supporting Information

for *Adv. Sci.*, DOI: 10.1002/advs.202001321

Extension of Compositional Space to the Ternary in Alloy Chiral Nanoparticles through Galvanic Replacement Reactions

Ziyue Ni,[#] Yuanmin Zhu,[#] Junjun Liu, Lin Yang, Peng Sun, Meng Gu, and Zhifeng Huang**

Supporting Information

Extension of Compositional Space to the Ternary in Alloy Chiral Nanoparticles through Galvanic Replacement Reactions

Ziyue Ni,[#] Yuanmin Zhu,[#] Junjun Liu, Lin Yang, Peng Sun, Meng Gu, and Zhifeng Huang**

Z. Y. Ni, Dr. J. J. Liu, Dr. L. Yang, P. Sun, Dr. Z. F. Huang
Department of Physics, Hong Kong Baptist University (HKBU), Kowloon Tong, Kowloon, Hong Kong SAR, China
Dr. Y. M. Zhu, P. Sun, Dr. M. Gu
Department of Materials Science and Engineering, Southern University of Science and Technology (SUSTech), Shenzhen 518055, China
E-mail: gum@sustech.edu.cn
Dr. Y. M. Zhu
SUSTech Academy for Advanced Interdisciplinary studies, Southern University of Science and Technology, Shenzhen 518055, China
Dr. J. J. Liu, Dr. L. Yang, Dr. Z. F. Huang
HKBU Institute of Research and Continuing Education, Shenzhen, Guangdong 518057, China
Dr. Z. F. Huang
Institute of Advanced Materials, State Key Laboratory of Environmental and Biological Analysis, Golden Meditech Centre for NeuroRegeneration Sciences, HKBU, Kowloon Tong, Kowloon, Hong Kong SAR, China
E-mail: zfhuang@hkbu.edu.hk

Table S1. Cathodic and anodic half-cell reactions with standard electrode potential (*E^0^*, versus standard hydrogen electrode or SHE) at 25^{0}C. The anodic reactions marked in gray do not occur in the GRR of Cu and Cu:M (M: Au, Ag, and TiO_2) CNPs (functioning as chiral sacrificial templates or CST) in a given electrolyte studied in this work.

Electrodes	Half-cell reaction $\boldsymbol{M^{m+}(aq) + me^- \leftrightharpoons M(s)}$	E^0 (V)	Electrolytes
**Cathode**	$Ag^+(aq) + e^- \rightarrow \boldsymbol{Ag(s)}$	+0.80	$AgNO_3$
	$[PtCl_4]^{2-}(aq) + 2e^- \rightarrow \boldsymbol{Pt(s)} + 4Cl^-(aq)$	+0.76	K_2PtCl_4
**Anode (CST)**	$AuCl_4^-(aq) + 3e^- \leftarrow \boldsymbol{Au(s)} + 4Cl^-(aq)$	+0.93	N. A.
	$Ti^{3+}(aq) + 2H_2O \leftarrow \boldsymbol{TiO_2(s)} + 4H^+ + e^-$	+0.762	
	$Cu^+(aq) + e^- \leftarrow \boldsymbol{Cu(s, CNP)}$	+0.52	
	$Cu^{2+}(aq) + 2e^- \leftarrow \boldsymbol{Cu(s, CNP)}$	+0.34	
	$AgCl(s) + e^- \leftarrow \boldsymbol{Ag(s)} + Cl^-(aq)$	+0.22	

S1. Calculation of the anisotropy *g*-factor

CD is the differential absorption of LCP and RCP, given by

$$CD = \Delta A = A_{LCP} - A_{RCP} \quad (S1)$$

where A_{LCP} and A_{RCP} are the absorption of LCP and RCP, respectively. According to Beer's law, ΔA, which is as a function of incident light wavelength, can be calculated by

$$\Delta A = \Delta\varepsilon Cl = (\varepsilon_{LCP} - \varepsilon_{RCP})Cl \quad (S2)$$

where ε_{LCP} and ε_{RCP} are the molar extinction coefficients for LCP and RCP light, respectively. C is the molar concentration, and l is the path length.

CD is usually measured in degrees of ellipticity θ, given by

$$\tan\theta = (E_R - E_L)/(E_R + E_L) \quad (S3)$$

where E_R and E_L are the magnitudes of the electric-field vectors of RCP and LCP, respectively. $\tan\theta$ is usually small and approximately equal to the angle θ in unit of radians. Given that the intensity of light, I, is proportional to the square of the electric-field vector, it can be derived

$$\theta \text{ (radians)} = (I_R^{1/2} - I_L^{1/2})/ (I_R^{1/2} + I_L^{1/2}) \quad (S4)$$

According to Beer's law,

$$I = I_0 \, e^{-A\ln 10} \quad (S5)$$

The ellipticity can then be written as,

$$\theta \text{ (radians)} = (e^{(\Delta A/2)\ln 10} - 1)/ (e^{(\Delta A/2)\ln 10} + 1) \quad (S6)$$

Because $\Delta A << 1$, after expanding the exponentials and neglecting the high-order terms, Eq. S6 leads to

$$\theta \text{ (degrees)} = \Delta A \, (\ln 10/4)(180/\pi) \quad (S7)$$

Combining Eq. S2 and S7,

$$\theta \text{ (mdeg)} = 32982\Delta\varepsilon Cl \quad (S8)$$

The anisotropy *g*-factor is defined by

$$g = 2(\Delta A/A) \quad (S9)$$

Combining Eq. S1, S8 and S9,

$$g = 2(\theta \text{ (mdeg)}/32982)/A \approx CD/(16500A) \quad (S10)$$

In this work, the samples were the close-packed array of CNPs deposited on sapphire, and the monitored A represents the extinction of the samples. As a result, Eq. 1 is obtained.

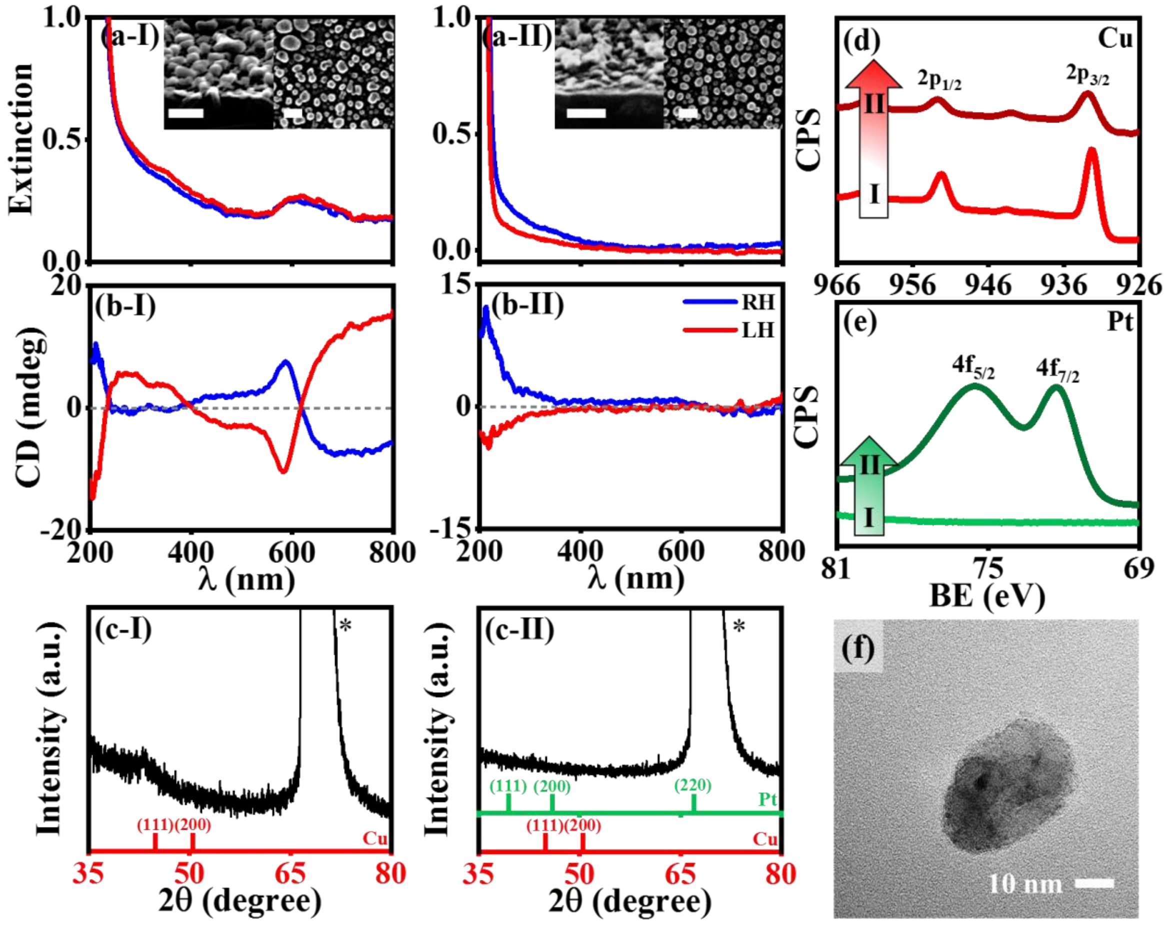

Figure S1. GRR of unary Cu CNPs in an aqueous electrolyte containing 10 μmol/L K_2PtCl_4, as a function of reaction duration (*t*): (I) 0, (II) 5 h. The Cu CNPs have the nominal *P* of ≈10 nm. The GRR was performed at room temperature through stirring the electrolyte at a rate of 380 rpm. UV/visible spectra: (a) extinction; (b) CD. (a, b) Blue lines: the GRR of RH-Cu CNPs; red lines: the GRR of LH-Cu CNPs. Insets: (a-I, a-II) SEM oblique (in the upper center) and top-down (in the upper right) images of the samples (scale bars: 100 nm). (c) XRD spectra of the Cu CNPs treated by the GRR as a function of *t*. The peaks marked by an asterisk are assigned to silicon on which the Cu CNPs were deposited. XPS spectra of the Cu CNPs treated with the GRR as a function of *t*: (d) Cu 2p; (e) Pt 4f. (d, e) Gradually darkening colors represent an elongation of the GRR from (I) 0 h to (II) 5 h. (f) TEM image of a Cu CNP treated by the GRR lasting for 5 h. Insets in (a-I, II) and (c-f): the GRR of the RH-Cu CNPs. **The 5-h GRR caused the generation of binary Cu:Pt nanoparticles without optical activity (b-II), indicating no chirality transfer from the Cu CNPs mediated with the GRR.**

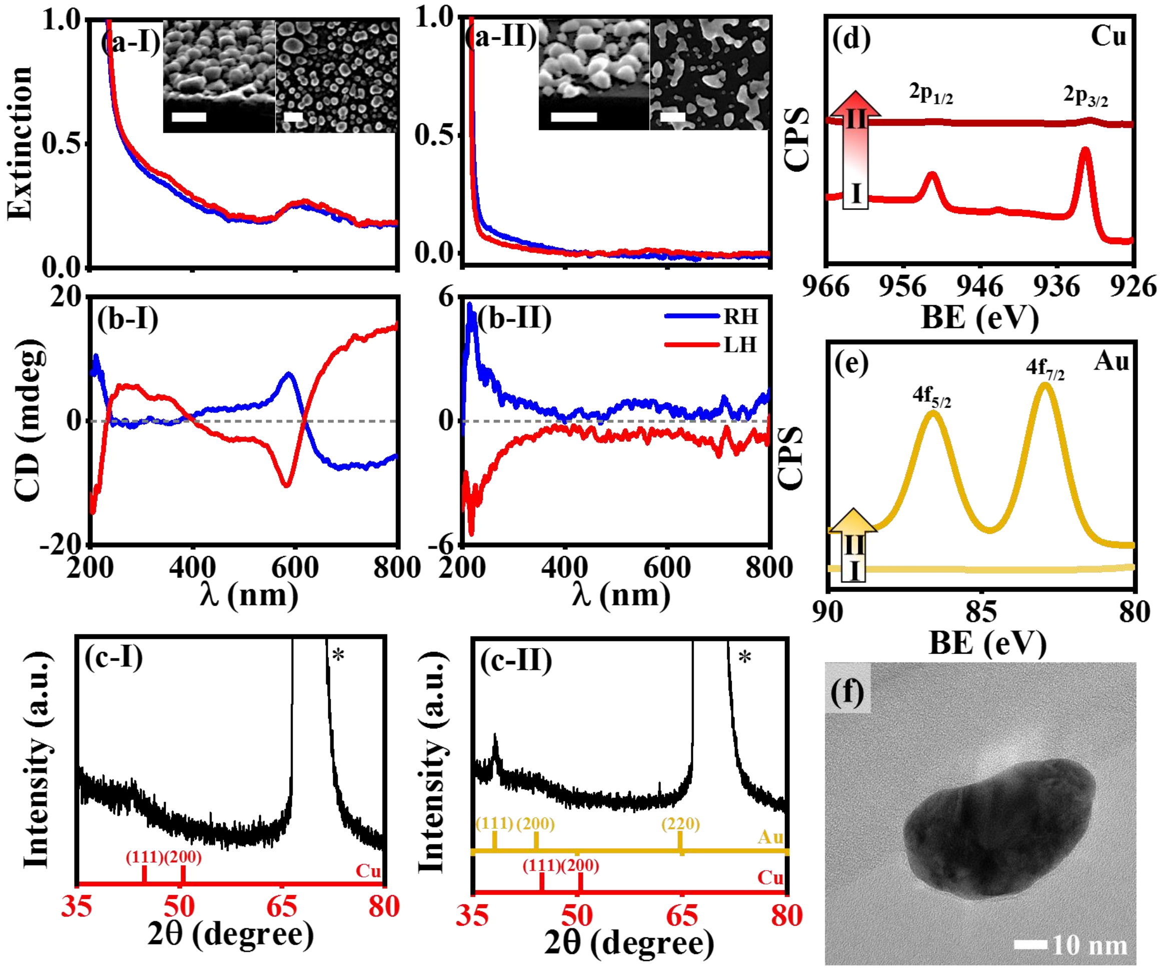

Figure S2. GRR of unary Cu CNPs in an aqueous electrolyte containing 10 μmol/L $HAuCl_4$, as a function of *t*: (I) 0, (II) 2 h. The Cu CNPs have the nominal *P* of ≈10 nm. The GRR was performed at room temperature through stirring the electrolyte at a rate of 380 rpm. UV/visible spectra: (a) extinction; (b) CD. (a, b) Blue lines: the GRR of RH-Cu CNPs; red lines: the GRR of LH-Cu CNPs. Insets: (a-I, a-II) SEM oblique (in the upper center) and top-down (in the upper right) images of the samples (scale bars: 100 nm). (c) XRD spectra of the Cu CNPs treated by the GRR as a function of *t*. The peaks marked by an asterisk are assigned to silicon on which the Cu CNPs were deposited. XPS spectra of the Cu CNPs treated by the GRR as a function of *t*: (d) Cu 2p; (e) Au 4f. (d, e) Gradually darkening colors represent an elongation of the GRR from (I) 0 h to (II) 2 h. (f) TEM image of a Cu CNP treated with the 2-h GRR. Insets in (a-I, II) and (c-f): the GRR of the RH-Cu CNPs. **The 2-h GRR caused the generation of binary Cu:Au nanoparticles with no optical activity (b-II), indicating no chirality transfer from the Cu CNPs mediated with the GRR.**

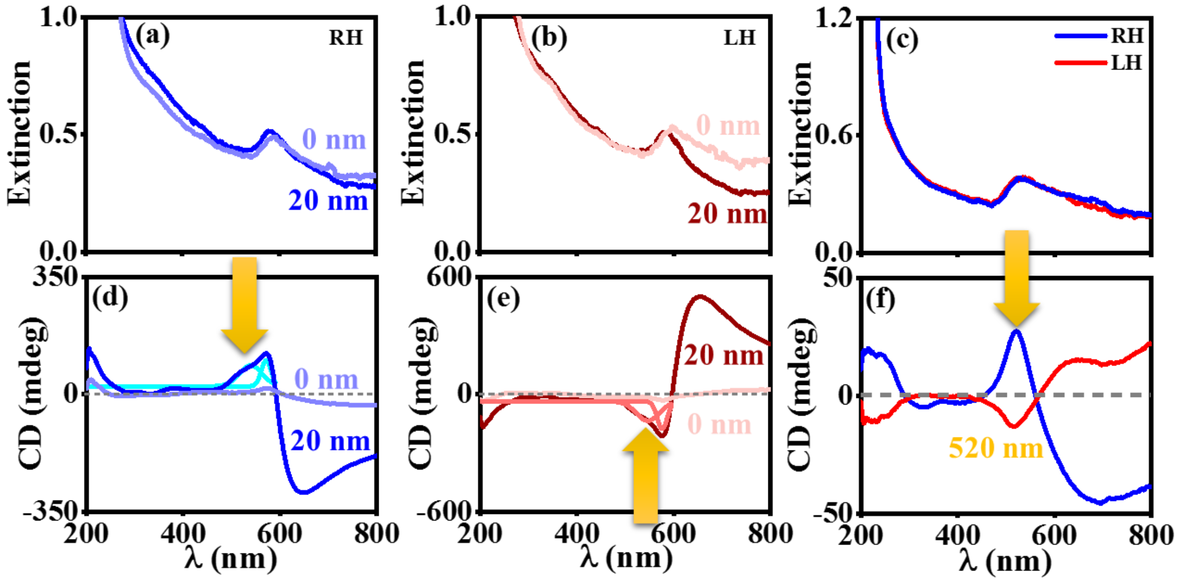

Figure S3. Chiroplasmonic induction of the dopant Au in binary Cu:Au CNPs. In the binary CNPs, the nominal *P* of the host Cu is ≈10 nm. (a, b, d, e) Cu:Au CNPs with a T_{Au} of 0 and 20 nm: (a, d) RH; (b, e) LH. (c, f) Unary Au CNPs with a nominal *P* of ≈10 nm and height (*H*) of ≈100 nm: RH (blue lines); LH (red lines). UV/visible spectra: (a-c) extinction; (d-f) CD. (d, e) Compared with the Cu CNPs (with a T_{Au} of 0 nm), the binary Cu:Au CNPs (with a T_{Au} of 20 nm) display an additional CD peak fitted at the wavelength of (d) 538 nm and (e) 544 nm, which are marked by yellow arrows. The additional CD peak is positive and negative in the RH- and LH-Cu:Au CNPs, respectively. (f) Unary Au CNPs exhibit a CD peak at the wavelength of ≈520 nm, with positive/negative sign for RH/LH, respectively. **The comparison of (d, e) with (f) illuminates the chirality transmission from the host Cu CNPs to the dopant Au, i.e., the chiroplasmonic induction of the dopant Au.** (c, f) The data have been published in Figure S5 (*Nano Lett.* 2019, 19, 7427). Copyright 2019, ACS.

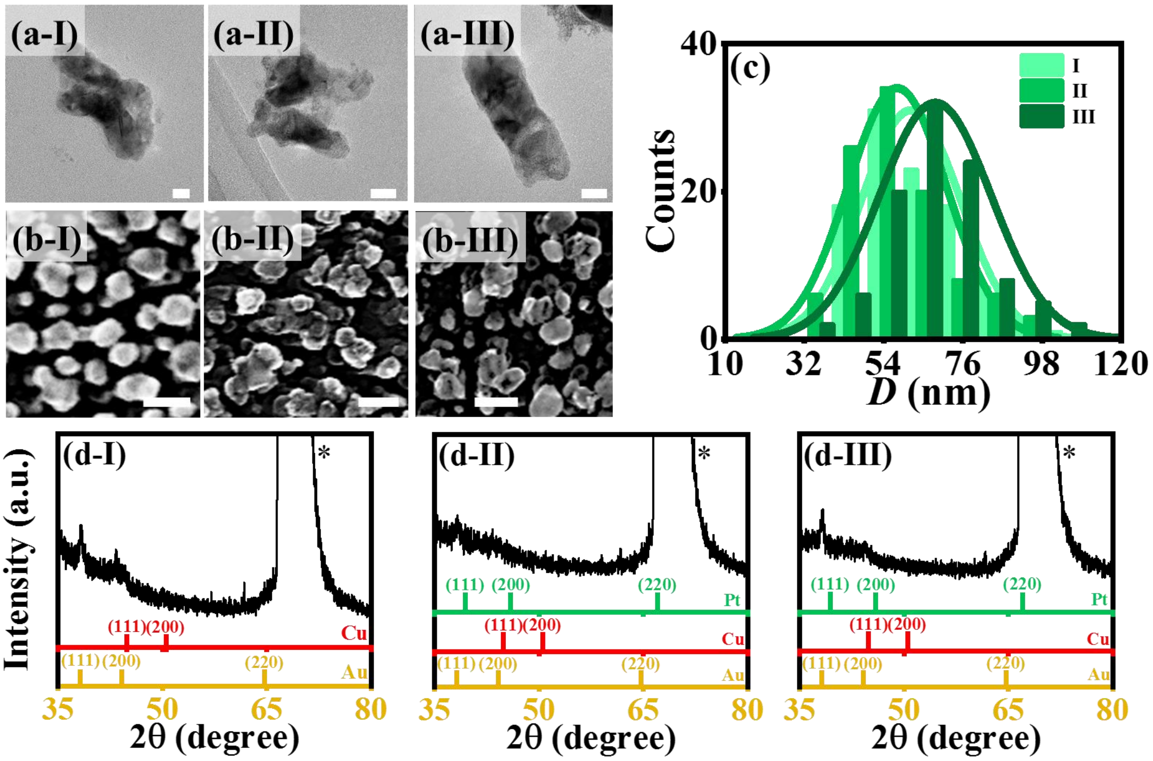

Figure S4. GRR of RH-binary Cu:Au CNPs in an aqueous electrolyte containing 10 μmol/L K_2PtCl_4 (Figure 1), as a function of *t*: (I) 0, (II) 5, and (III) 9 h. (a) TEM images of the samples (scale bars: 20 nm). (b) SEM top-down images of the samples (scale bars: 100 nm). (c) Histograms of the diameter *D* (monitored with the SEM top-down images) of the CNPs formed by the GRR of the Cu:Au CNPs as a function of *t*, fitted with the normal distribution. *D* was statistically evaluated to be (I) 62 ± 14 nm, (II) 58 ± 14 nm, and (III) 68 ± 16 nm. (d) XRD spectra of the Cu:Au CNPs treated by the GRR as a function of *t*. The peaks marked by an asterisk are assigned to silicon on which the Cu:Au CNPs were deposited.

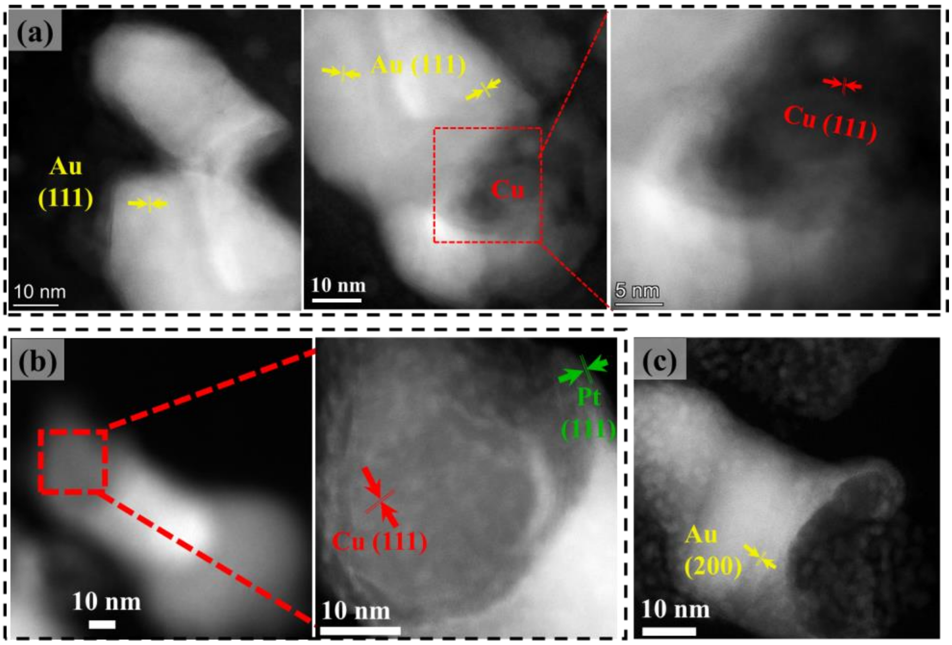

Figure S5. HRTEM image of RH-binary Cu:Au CNPs treated with the GRR, in an aqueous electrolyte containing 10 µmol/L K_2PtCl_4 (Figure 1), as a function of *t*: (a) 0, (b) 5, and (c) 9 h.

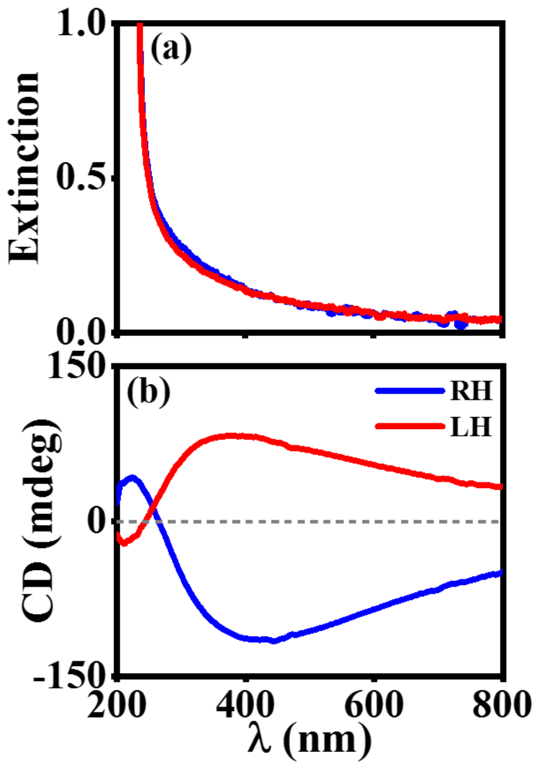

Figure S6. GLAD of unary Pt CNPs, having the nominal *P* of ≈10 nm and *H* of ≈50 nm. UV/visible spectra: (a) extinction; (b) CD. Blue lines: RH; red lines: LH.

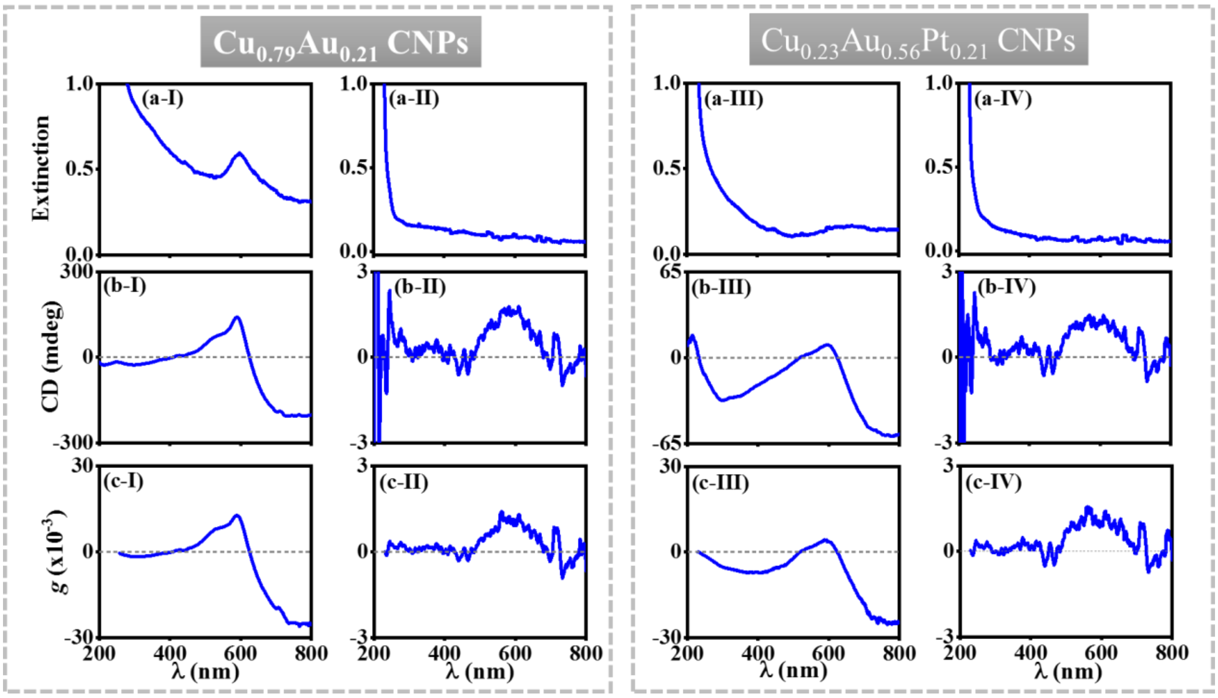

Figure S7. Optical activity of (I, II) the RH-binary Cu:Au CNPs and (III, IV) the RH-ternary Cu:Au:Pt CNPs, (I, III) vertically protruding on sapphires and dispersed in DI water. UV/visible spectra: (a) extinction; (b) CD; (c) anisotropic *g*-factor. To well disperse the CNPs, the CNPs were modified with surfactant cetrimonium bromide (CTAB) and then ultrasonicated in DI water. However, the CNP aggregation could not be effectively prevented, so that the dispersed CNPs showed very weak CD signals.

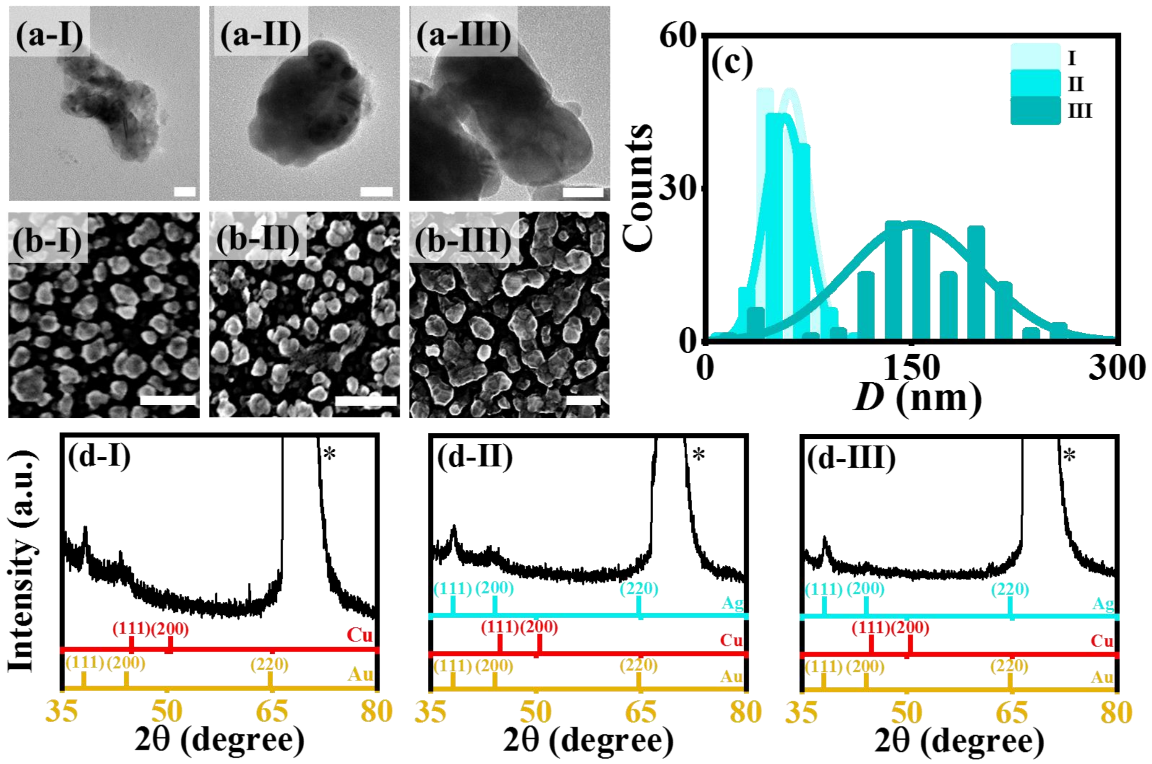

Figure S8. GRR of RH-binary Cu:Au CNPs in an aqueous electrolyte containing 20 μmol/L $AgNO_3$ (Figure 2), as a function of *t*: (I) 0, (II) 2, and (III) 7 h. (a) TEM images of the samples (scale bars: 25 nm). (b) SEM top-down images of the samples (scale bars: 200 nm). (c) Histograms of the diameter *D* (monitored with the SEM top-down images) of the CNPs formed through the GRR of the binary CNPs as a function of *t*, fitted with the normal distribution. *D* was statistically evaluated to be (I) 62 ± 14 nm, (II) 58 ± 15 nm, and (III) 152 ± 48 nm. (d) XRD spectra of the binary CNPs treated by the GRR as a function of *t*. The peaks marked by an asterisk are assigned to silicon on which the Cu:Au CNPs were deposited.

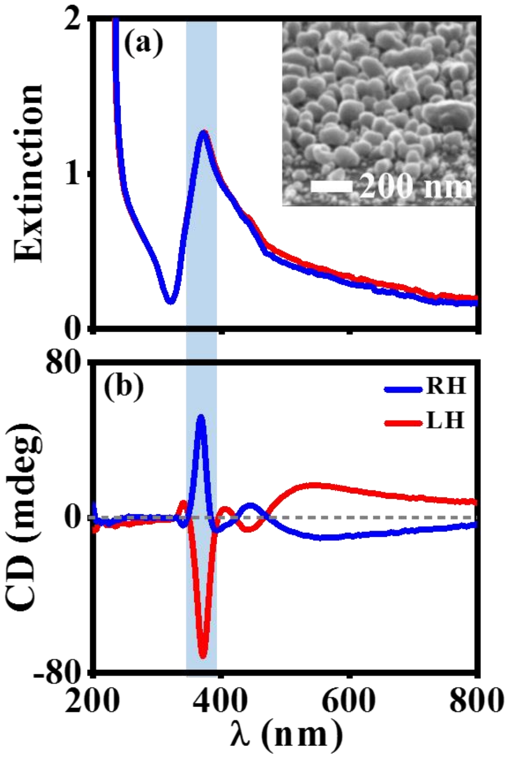

Figure S9. GLAD of unary Ag CNPs, having the nominal *P* of ≈10 nm and *H* of ≈100 nm. UV/visible spectra: (a) extinction; (b) CD. (a, b) Blue lines: RH; red lines: LH. The transverse chiroplasmonic mode of Ag is highlighted by sky blue background. Inset: (a) SEM tilted image of LH-Ag CNPs.

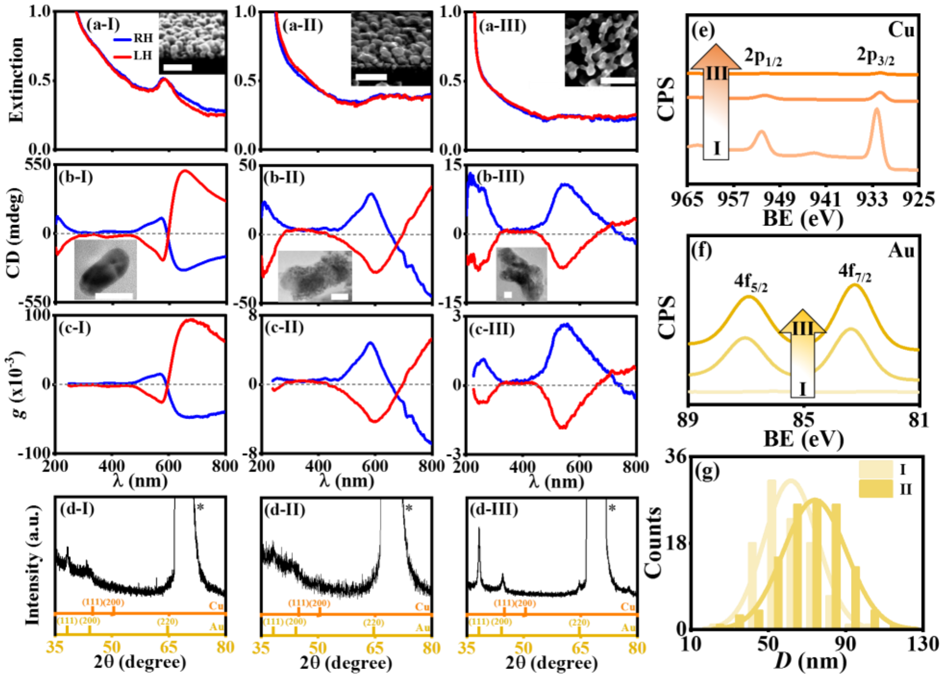

Figure S10. GRR of binary Cu:Au CNPs in an aqueous electrolyte containing 10 µmol/L $HAuCl_4$, as a function of *t*: (I) 0, (II) 1, and (III) 3 h. In the binary CNPs, the nominal *P* of the host Cu is ≈10 nm and T_{Au} is 20 nm. The GRR was performed at room temperature through stirring the electrolyte at a rate of 380 rpm. UV/visible spectra: (a) extinction; (b) CD; (c) anisotropic *g*-factor. (a-c) Blue lines: the GRR of RH-Cu:Au CNPs; red lines: the GRR of LH-Cu:Au CNPs. Insets: SEM (a-I, a-II) oblique and (a-III) top-down images of the samples (scale bars: 200 nm); (b-I, b-II, b-III) TEM images of the samples (scale bars: 20 nm). (d) XRD spectra of the binary CNPs treated with the GRR as a function of *t*. The peaks marked by an asterisk are assigned to silicon on which the Cu:Au CNPs were deposited. XPS spectra of the Cu:Au CNPs treated with the GRR as a function of *t*: (e) Cu 2p; (f) Au 4f. (e, f) Gradually darkening colors represent an elongation of the GRR from (I) 0 h to (III) 3 h. (g) Histograms of the diameter *D* (monitored with the SEM top-down images) of the CNPs formed by the GRR of the binary CNPs as a function of *t*, fitted with the normal distribution. *D* was statistically evaluated to be (I) 62 ± 14 nm, and (II) 74 ± 16 nm. The 3 h-GRR caused the CNPs to aggregate together (inset in a-III), preventing from the statistical evaluation of *D*. Insets in (a-I, II, III; b-I, II, III) and (d-g): the GRR of the RH-binary CNPs. **Elongating the GRR led to the decrease of the Cu at% (e) and the increase of the Au at% (f), and the 3 h-GRR enabled the generation of the binary CNPs having optical activity (c-III). It is illustrated that the dopant Au assist the GRR-mediated chirality transmission from the host Cu CNPs to Au.**

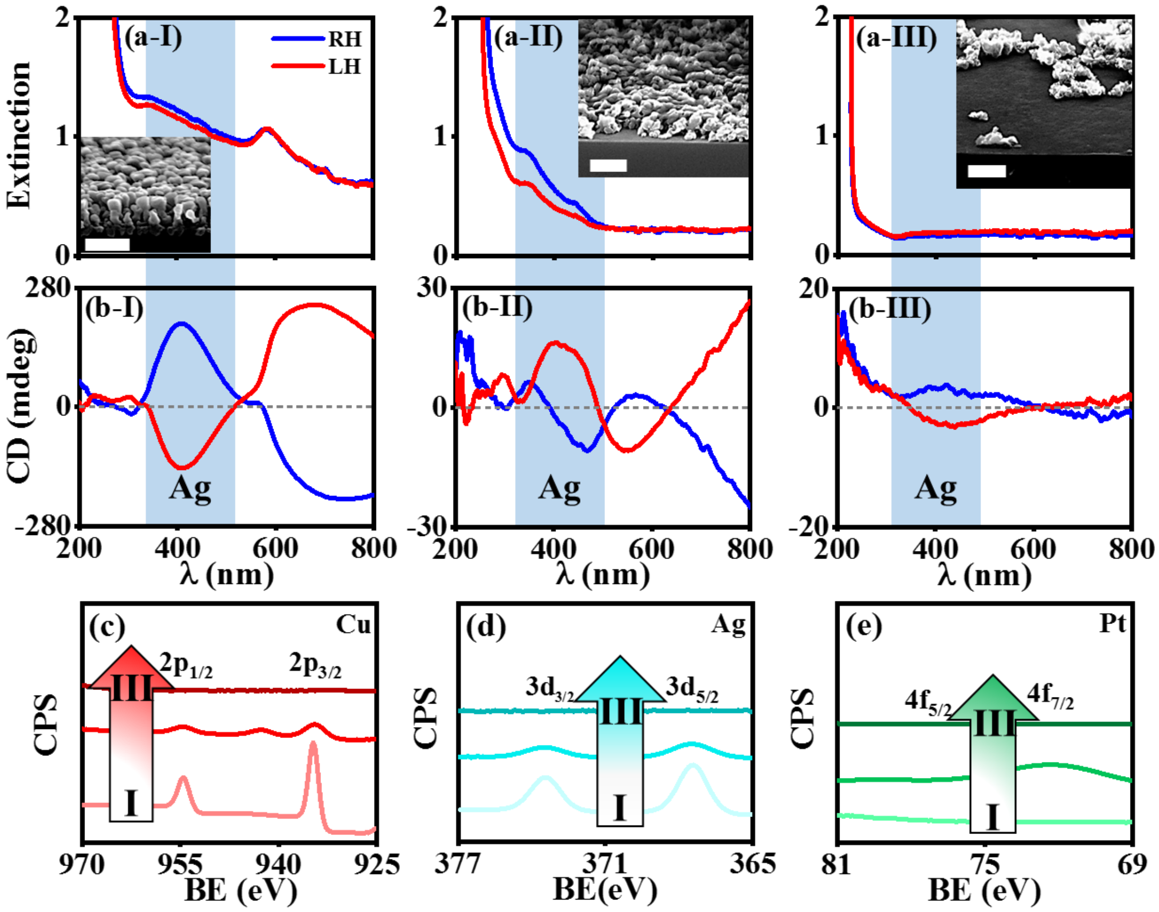

Figure S11. GRR of binary Cu:Ag CNPs in an aqueous electrolyte containing 10 μmol/L K_2PtCl_4, as a function of *t*: (I) 0, (II) 20 min, and (III) 1 h. In the binary CNPs, the nominal *P* of the host Cu is ≈10 nm and T_{Ag} is 20 nm. The GRR was performed at room temperature, through stirring the electrolyte at a rate of 380 rpm. UV/visible spectra: (a) extinction; (b) CD. (a-c) Blue lines: the GRR of RH-Cu:Ag CNPs; red lines: the GRR of LH-Cu:Ag CNPs. Sky blue backgrounds are used to mark the LSPR and CD signals of the dopant Ag. Insets: (a-I, a-II, a-III) SEM oblique images of the samples (scale bars: 200 nm). XPS spectra of the Cu:Ag CNPs treated with the GRR as a function of *t*: (c) Cu 2p; (d) Ag 3d; (e) Pt 4f. (c-e) Gradually darkening colors represent an elongation of the GRR from (I) 0 h to (III) 1 h. Insets in (a-I, II, III) and (c-e): the GRR of the RH-binary CNPs. **The 1-h GRR caused the nano-products to show very weak optical activity (b-III), indicating no effective chirality transfer from the host Cu CNPs mediated with the GRR.**

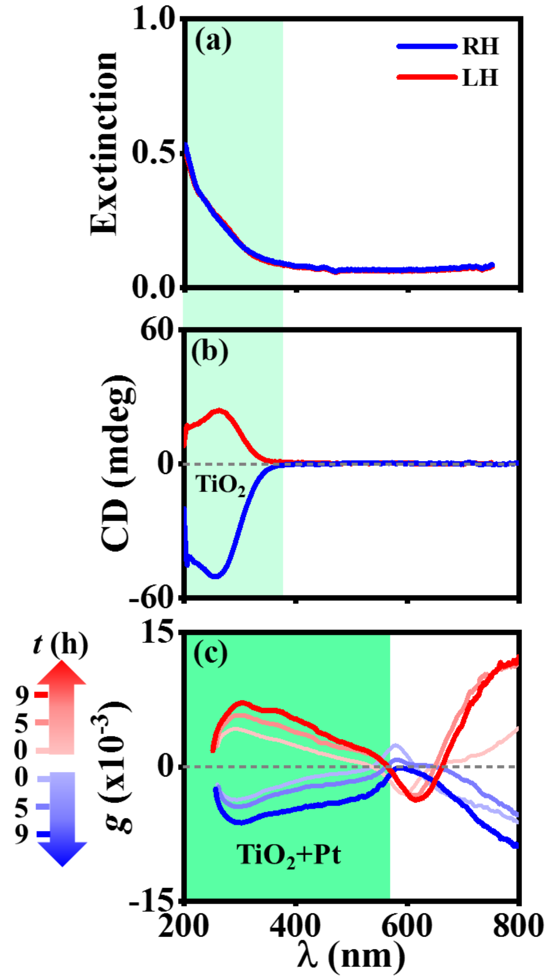

Figure S12. GLAD of TiO_2 CNPs, having the nominal *P* of ≈10 nm and *H* of ≈50 nm. UV/visible spectra: (a) extinction; (b) CD. (a, b) Blue lines: RH; red lines: LH. The transverse chiroplasmonic mode of TiO_2 is highlighted by light green background. (c) Overlay of (c-I, II, III) in **Figure 3**. The transverse chiroptical mode of TiO_2 and the precipitating Pt is highlighted by green background.

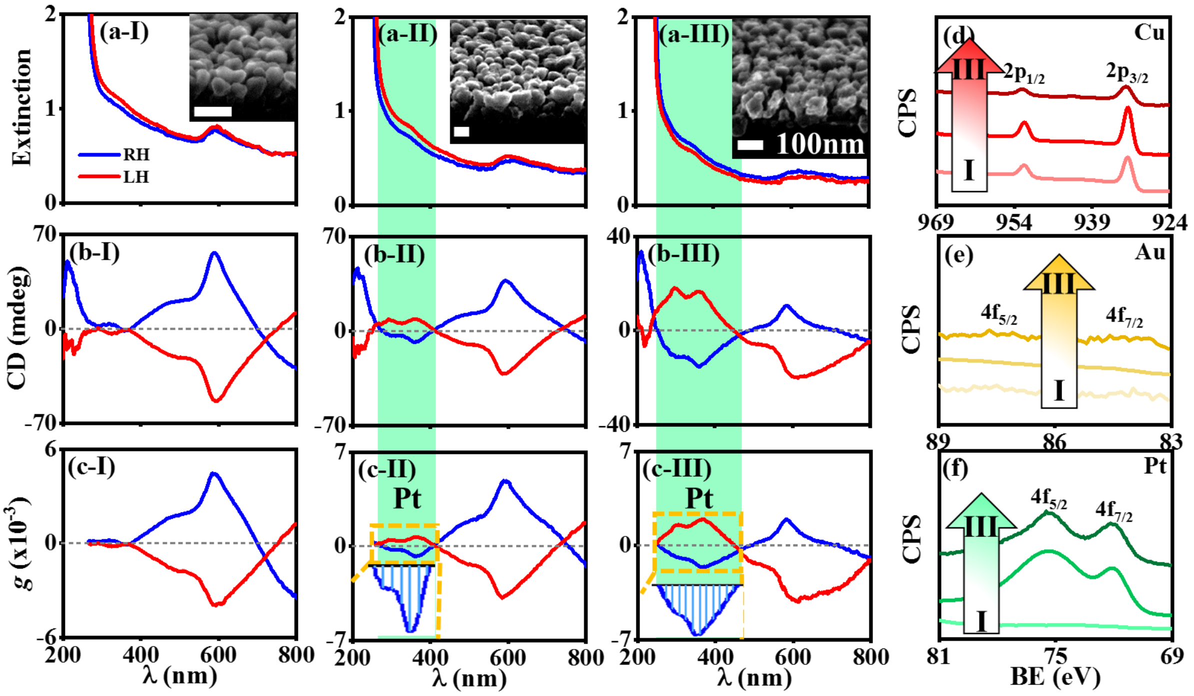

Figure S13. GRR of binary Cu:Au CNPs (T_{Au} = 2 nm) in an aqueous electrolyte containing 10 μmol/L K_2PtCl_4, as a function of *t*: (I) 0, (II) 5, and (III) 9 h. The GRR was performed at room temperature through stirring the electrolyte at a rate of 380 rpm, to generate ternary Cu:Au:Pt CNPs. UV/visible spectra: (a) extinction; (b) CD; (c) anisotropic *g*-factor. (a-c) Blue lines: the GRR of RH-Cu:Au CNPs; red lines: the GRR of LH-Cu:Au CNPs. Green backgrounds are used to mark the chiroplasmonic signals of the precipitated Pt. Insets: (a-I, a-II, a-III) SEM oblique images of the samples (scale bars: 100 nm); (c-II, c-III) the calculation of the integrated area of the anisotropic *g*-factor peaks highlighted with orange dash squares. XPS spectra of the Cu:Au CNPs treated with the GRR as a function of *t*: (d) Cu 2p; (e) Au 4f; (f) Pt 4f. (d-f) Gradually darkening colors represent an elongation of the GRR from (I) 0 h to (III) 9 h. Insets in (a-I, II, III) and (d-f): the GRR of the RH-binary CNPs.

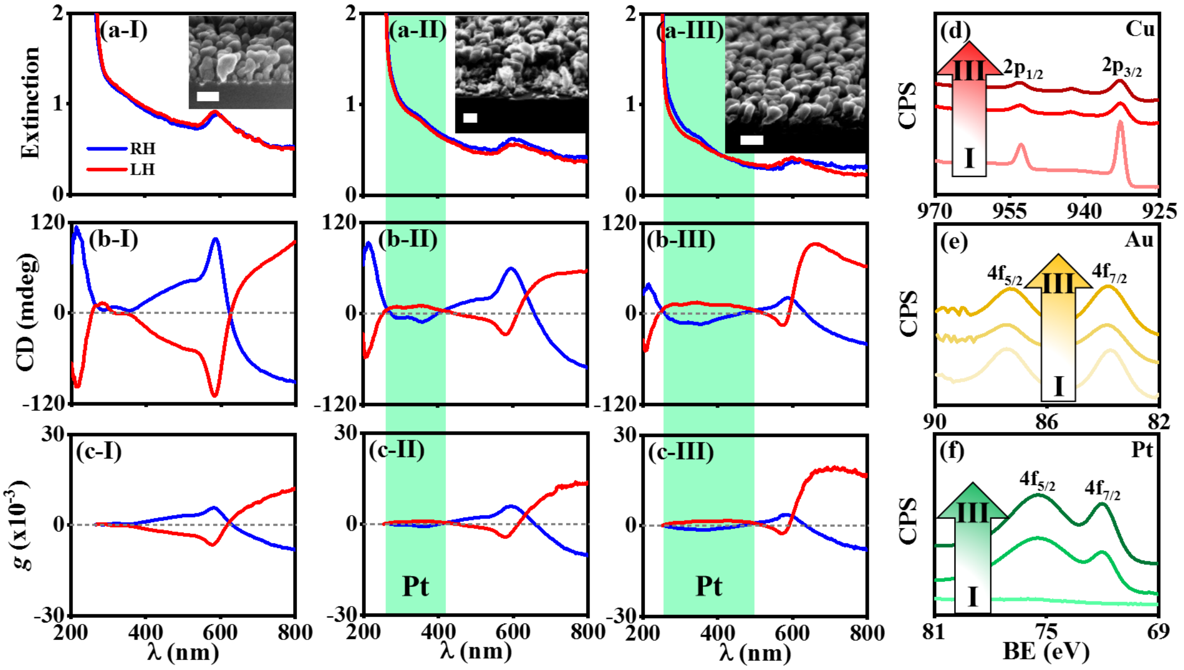

Figure S14. GRR of binary Cu:Au CNPs (T_{Au} = 10 nm) in an aqueous electrolyte containing 10 µmol/L K_2PtCl_4, as a function of *t*: (I) 0, (II) 5, and (III) 9 h. The GRR was performed at room temperature through stirring the electrolyte at a rate of 380 rpm, to generate ternary Cu:Au:Pt CNPs. UV/visible spectra: (a) extinction; (b) CD; (c) anisotropic *g*-factor. (a-c) Blue lines: the GRR of RH-Cu:Au CNPs; red lines: the GRR of LH-Cu:Au CNPs. Green backgrounds are used to mark the chiroplasmonic signals of the precipitated Pt. Insets: (a-I, a-II, a-III) SEM oblique images of the samples (scale bars: 100 nm). XPS spectra of the binary CNPs treated by the GRR as a function of *t*: (d) Cu 2p; (e) Au 4f; (f) Pt 4f. (d-f) Gradually darkening colors represent an elongation of the GRR from (I) 0 h to (III) 9 h. Insets in (a-I, II, III) and (d-f): the GRR of the RH-binary CNPs.

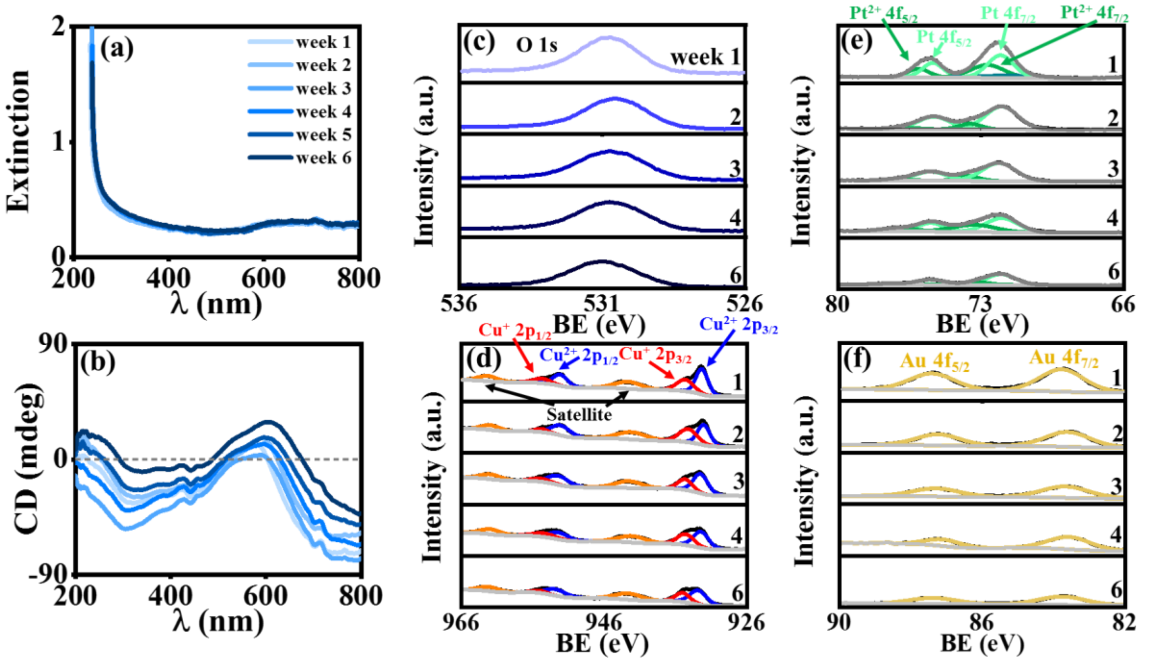

Figure S15. Ambient aging of the ternary RH-$Cu_{0.85}Au_{0.13}Pt_{0.02}$ CNPs (Figure 5a, b). UV/visible spectra of (a) Extinction and (b) CD, as a function of aging duration. XPS spectra as a function of aging duration: (c) O1s; (d) Cu2p; (e) Pt4f; (f) Au4f.

Note that the XPS peaks of all the metallic elements tend to decrease in amplitude with the ambient aging (Figure S15d–f and Figure S16d–f). It is well known that XPS has the detection depth < 10 nm, so that the continuous oxidation at the CNP surfaces causes the shrinkage of the metallic at% within the detection depth, accounting for the above-mentioned phenomena in the XPS spectra. Furthermore, the composition of the ternary $Cu_{0.85}Au_{0.13}Pt_{0.02}$ and $Cu_{0.23}Au_{0.56}Pt_{0.21}$ CNPs was evaluated using the EDS mapping, which enables the detection of the entire composition of the CNPs. In contrast, the results shown in Figure 5a, c were evaluated using XPS that only provides the surface composition. As a result, the XPS-monitored at% ratio at the surfaces during the oxidation deviates from the original composition of the ternary CNPs characterized using the EDS mapping.

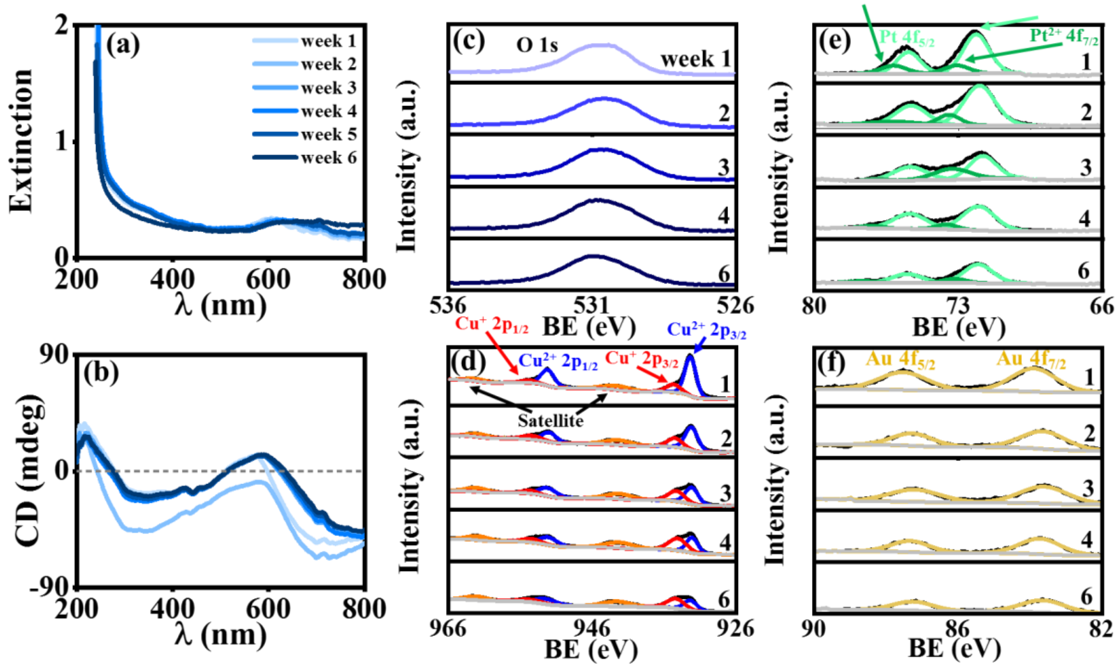

Figure S16. Ambient aging of the ternary RH-$Cu_{0.23}Au_{0.56}Pt_{0.21}$ CNPs (Figure 5c, d). UV/visible spectra of (a) Extinction and (b) CD, as a function of aging duration. XPS spectra as a function of aging duration: (c) O1s; (d) Cu2p; (e) Pt4f; (f) Au4f.

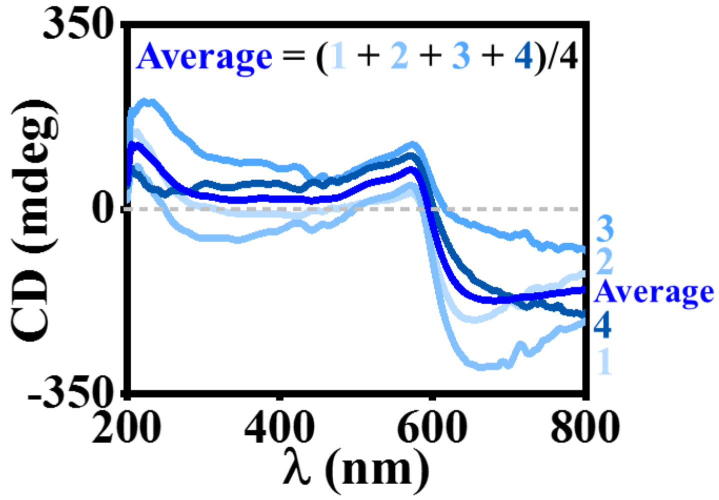

Figure S17. CD Characterization of the host $Cu_{0.79}Au_{0.21}$ CNPs. Four CD spectra (spectrum 1–4) were subsequently recorded, and after monitoring each CD spectrum, the sample was manually rotated in an angle of 90° around its normal axis before measuring the next CD spectrum. Then the four CD spectra were algebraically averaged to obtain a CD spectrum of the sample, to eliminate the linear birefringence.
